# Safety of a Novel *Listeria monocytogenes*-Based Vaccine Vector Expressing NcSAG1 (*Neospora caninum* Surface Antigen 1)

**DOI:** 10.3389/fcimb.2021.675219

**Published:** 2021-08-25

**Authors:** William Robert Pownall, Dennis Imhof, Nerea Fernandez Trigo, Stephanie C. Ganal-Vonarburg, Philippe Plattet, Camille Monney, Franck Forterre, Andrew Hemphill, Anna Oevermann

**Affiliations:** ^1^ Division of Small Animal Surgery, Department of Clinical Veterinary Sciences, Vetsuisse Faculty, University of Bern, Bern, Switzerland; ^2^ Institute of Parasitology, DIP, Vetsuisse Faculty, University of Bern, Bern, Switzerland; ^3^ Department for BioMedical Research (DBMR), Universitätsklinik für Viszerale Chirurgie und Medizin, Inselspital, Bern University Hospital, University of Bern, Bern, Switzerland; ^4^ Division of Neurological Sciences, DCR-VPH, Vetsuisse Faculty, University of Bern, Bern, Switzerland

**Keywords:** microbial vaccine, *Listeria monocytogenes*, neosporosis, *Neospora caninum*, immune response, *in vitro* safety, *in vivo* safety

## Abstract

*Listeria monocytogenes* (LM) has been proposed as vaccine vector in various cancers and infectious diseases since LM induces a strong immune response. In this study, we developed a novel and safe LM-based vaccine vector platform, by engineering a triple attenuated mutant (Lm3Dx) (ΔactA, ΔinlA, ΔinlB) of the wild-type LM strain JF5203 (CC 1, phylogenetic lineage I). We demonstrated the strong attenuation of Lm3Dx while maintaining its capacity to selectively infect antigen-presenting cells (APCs) *in vitro*. Furthermore, as proof of concept, we introduced the immunodominant *Neospora caninum* (Nc) surface antigen NcSAG1 into Lm3Dx. The NcSAG1 protein was expressed by Lm3Dx_SAG1 during cellular infection. To demonstrate safety of Lm3Dx_SAG1 *in vivo*, we vaccinated BALB/C mice by intramuscular injection. Following vaccination, mice did not suffer any adverse effects and only sporadically shed bacteria at very low levels in the feces (<100 CFU/g). Additionally, bacterial load in internal organs was very low to absent at day 1.5 and 4 following the 1^st^ vaccination and at 2 and 4 weeks after the second boost, independently of the physiological status of the mice. Additionally, vaccination of mice prior and during pregnancy did not interfere with pregnancy outcome. However, Lm3Dx_SAG1 was shed into the milk when inoculated during lactation, although it did not cause any clinical adverse effects in either dams or pups. Also, we have indications that the vector persists more days in the injected muscle of lactating mice. Therefore, impact of physiological status on vector dynamics in the host and mechanisms of milk shedding requires further investigation. In conclusion, we provide strong evidence that Lm3Dx is a safe vaccine vector in non-lactating animals. Additionally, we provide first indications that mice vaccinated with Lm3Dx_SAG1 develop a strong and Th1-biased immune response against the Lm3Dx-expressed neospora antigen. These results encourage to further investigate the efficiency of Lm3Dx_SAG1 to prevent and treat clinical neosporosis.

## Introduction

In both human and veterinary medicine, the implementation of vaccines revealed to be one of the most effective strategies to prevent morbidity and mortality from infectious diseases. Case in points include small-pox (Meyer et al., 2020), rabies (Fischer and Schnell, 2018), polio, mumps, measles, rubella, varicella (Di Pietrantonj et al., 2020), meningococcal infections, Ebola (Henao-Restrepo et al., 2017), myxomatosis, canine distemper, parvovirosis, herpesvirus infections, and leptospirosis (Davis-Wurzler, 2014; Afghah et al., 2017; Jiang et al., 2019). Currently, many marketed vaccines rely on live-attenuated or killed/inactivated pathogens. For improved safety and immunogenicity, significant efforts have been made to develop alternative approaches using subunit-, DNA-, mRNA- or vector-based vaccines, even more so in the past months in the light of the COVID-19 pandemic (Griot et al., 2004; Nielsen et al., 2012; Fukuhara et al., 2016; Sharma et al., 2020).

Although to this day quantification of induced antibodies is used to evaluate immunogenicity of vaccines, it is increasingly recognized that T-cell responses are instrumental for a protective and sustained immunity against numerous infectious diseases (Gilbert, 2011; Gray et al., 2018; Jameson and Masopust, 2018; Peng et al., 2020; Sharma et al., 2020). Indeed, humoral responses proved insufficient in the control of certain pathogens (Fischer and Schnell, 2018; Vetter et al., 2018) and there is still an unmet need for vaccines against diseases such as tuberculosis, HIV (Gray et al., 2018) or protozoan parasites such as *Toxoplasma gondii* and the closely related *Neospora caninum* (Nc) (Leong et al., 2009; Wang et al., 2014).

Nc is an obligatory intracellular protozoan parasite belonging to the phylum apicomplexa and a major infectious cause of abortion and reproductive failure in cattle and small ruminants due to transplacental passage of rapidly proliferating tachyzoites and subsequent infection of the fetus (Schares et al., 1998; Moreno et al., 2012). Economic losses in the global cattle industry due to Nc-associated abortion and premature culling are estimated to amount up to 1.3 billion US $ yearly (Reichel et al., 2013). Models predict vaccination to be the most cost-effective approach to reduce abortions and reproductive failure in cattle herds with high Nc prevalence (Liu et al., 2020). Additionally, vaccines might prevent severe neuromuscular disease (Anvari et al., 2020) and fecal oocyst shedding in infected dogs, which are a major source of exogenous transmission to cattle (McAllister et al., 1998; Monney et al., 2011). However, despite strong efforts of the researcher community, no commercial vaccine is currently available.

Most vaccine trials provided evidence that a Th1-biased or a balanced Th1/Th2 immune response offer protection against horizontal and vertical transmission because they simulate the immune response during Nc infection that is associated with a robust development of specific CD4^+^ and CD8^+^ T-cells targeting the intracellular stage of the parasite (Hemphill et al., 2006; Aguado-Martínez et al., 2019). In contrast, vaccines triggering only a humoral immune response proved to be insufficient in preventing the disease because antibodies act only against the extracellular stage (Monney et al., 2011; Hemphill et al., 2016).

Over the last decades, *Listeria monocytogenes* (Lm), a gram-positive and facultatively intracellular bacterium, has evolved to a paradigmatic pathogen for immunology research (Cossart and Lebreton, 2014). Lm infection generates strong innate and adaptive immune responses and, thus, Lm has been increasingly recognized as a vaccine vector for the prevention and therapy of tumors and infectious diseases showing promising results in clinical trials (Liu, 2006; Singh and Wallecha, 2011; Wood and Paterson, 2014; Flickinger et al., 2018; Wendel Naumann and Leath, 2020).

Lm is an opportunistic pathogen that may cause serious disease including gastroenteritis, septicemia, abortions and neurolisteriosis in humans and animals (Schlech, 2019; Matle et al., 2020). Virulence of Lm is essentially governed by a defined set of crucial virulence factors regulating cellular invasion and intercellular spread. Invasion of most cells is mediated by the bacterial surface proteins InlA and InlB that bind cellular receptors present on a wide range of host cells and induce bacterial endocytosis (Lecuit et al., 1997). However, phagocytosis of Lm by professional phagocytes including antigen-presenting cells (APCs) is independent of theses receptors. Once internalized, Lm is either processed by the phagolysosome, or escapes the phagosome prior to phagolysosomal fusion and enters the cytosol, a process dependent of proteins encoded by *hly, plcA* and *plcB* (Radoshevich and Cossart, 2018). Once in the cytosol, Lm relies on an actin-assembly inducing protein encoded by the *actA* gene, to highjack the cellular actin machinery enabling Lm to spread from cell to cell (Radoshevich and Cossart, 2018).

During infection, Lm triggers potent innate and adaptive immune responses when phagocytosed by APCs (Flickinger et al., 2018; D’Orazio, 2019). Importantly, proteins from bacteria that reach the cytosol of infected APCs are processed and loaded on major histocompatibility complex (MHC) class I molecules for presentation to CD8^+^ cytotoxic T-cells (Ikonomidis et al., 1994; Pamer, 2004; Flickinger et al., 2018; Chávez Arroyo and Portnoy, 2020; Zeng et al., 2020), while bacterial proteins that are degraded within phagolysosomes of APCs are delivered to MHC class II associated epitope presentation for CD4^+^ T-cell priming (D’Orazio, 2019).

By deleting one or more crucial virulence genes, Lm virulence is easily and significantly attenuated preventing systemic infection of the host, while immunogenicity is maintained (Brockstedt et al., 2004). Thus, attenuated Lm is a promising vaccine vector to be used against diseases including neosporosis, for which a Th1-mediated immune response is required to confer protection (Ikonomidis et al., 1994; Brockstedt et al., 2004; Johnson et al., 2011; Wood and Paterson, 2014; Flickinger et al., 2018; D’Orazio, 2019; Chávez Arroyo and Portnoy, 2020). Additionally, the Lm vector has the advantage over other microbial vectors to overcome pre-existent or vector-induced immunity in the host allowing homologous prime-boost vaccination schemes without compromising its efficacy in T-cell priming (Leong et al., 2009).

The aim of our study was to develop an attenuated Lm vaccine vector (Lm3Dx) platform that is safe and expresses foreign antigen. To demonstrate proof of concept for triggering of a Th1 immune response we selected the NcSAG1 as antigen, which is a highly immunogenic protein, expressed on the surface of Nc tachyzoites and implicated to have a functional role in host cell invasion (Hemphill et al., 1997).

## Material And Methods

### Experimental Setup

Additionally to the crucial virulence genes *actA*, *inlA*, *inlB*, the fosfomycin resistance gene (*fosX*) was deleted from the parental JF5203 strain (ST1, CC1) (Aguilar-Bultet et al., 2018). *FosX* encodes a protein involved in inherent fosfomycin resistance of Lm outside the host (Scortti et al., 2018), and deletion renders the vaccine vector highly sensitive to this antibiotic and selectable without introducing exogenous antibiotic resistance genes while cloning. The resulting Lm vector Lm3Dx was first tested for genetic stability upon serial passaging and for infection dynamics in canine cell lines as surrogates for epithelial cells and APCs. The gene coding for the major Nc surface antigen NcSAG1 (*sag1*) was fused to the first 300 nucleotides of *actA* containing the signal peptide and PEST sequences to enhance antigen processing (Sewell et al., 2008) and was inserted into Lm3Dx *via* homologous recombination to avoid the introduction of exogenous antibiotic resistance genes. Homologous recombination, as opposed to plasmid integration methods used for other Lm-based vaccine vectors, does not require growth of the engineered vector on selective media containing antibiotics (Sacco et al., 2016) to ensure retention of the antigen encoding genes, thereby conferring high safety and stability to our vaccine strain. Bacterial NcSAG1 expression by the engineered Lm3Dx-NcSAG1 was confirmed by real-time PCR (RT-PCR) and Western Blot (WB) before performing *in vivo* safety and immunogenicity testing. For the latter, female pregnant and non-pregnant BALB/c mice were vaccinated intramuscularly (i.m.) with Lm3Dx expressing NcSAG1 using a homologous prime-boost vaccination scheme. In mice, bacterial spread to organs and to newborn pups, fecal and milk shedding (**Table 1**), genetic stability of isolated Lm3Dx_NcSAG1 and indicators of humoral and cellular immune response to NcSAG1 were evaluated.

**Table 1:** Scheme of the vaccination and sampling protocol performed for each mouse experiment.

Day	-3	-2	-1	0	1	2	3	4	5	6	7	8	9	10	11	12	13	14	15	16	17	18	19	20	21	22	23	24	25	26	27	28	29	30	31	32	33	34	35	36	37	38	39	40	41	42	43	44	45	46	47	48	49	50	51	52	53-73	74
***In-vivo* safety**																																																										
**Lm3Dx**																																																										
Immunization				X														X														X																										
Fecal sampling			X	X	X	X		X	X	X	X			X			X	X	X	X	X	X	X	X	X			X			X	X	X	X	X	X	X	X	X	X			X			X												
Serum sampling			X															X														X														X												
Euthanasia and organ sampling						X																																								X												
Splenocyte stimulation																																														X												
**WT**																																																										
Inoculation				X																																																						
Fecal sampling			X	X	X	X																																																				
Euthanasia and organ sampling						X																																																				
***Milk excretion***																																																										
Immunization				X																																																						
Milk sampling					X	X	X	X																																																		
Euthanasia, organ sampling								X																																																		
***Birth Interference***																																																										
Immunization				X														X														X																										
Fecal sampling	X				X	X		X			X								X	X		X			X								X	X		X			X				X			X												
Mating																													X	X	X																											
Pups birth																																																					X	X	X	X		
Serum sampling	X																																																									X
Euthanasia, organ sampling, splenocyte stimulation																																																										X

### Generation and Quality Control of the Vaccine Vector

#### Bacterial Strains, Plasmids, and Primers

Bacterial strains and plasmids used in this study are listed in **Supplementary Files S2**, **S3** and primers in **Supplementary File S4**, respectively.

The pMADΔactA plasmid used to remove *actA* was previously described (Henke et al., 2015). To generate the pHOSSΔinlAB plasmid, flanking regions of the *inlAB* region including the first 12 N-terminal nucleotides of *inlA* and the last 36 C-terminal nucleotides of *inlB* were amplified with GoTaq^®^DNA polymerase (Promega Corporation, Madison, USA) using the primer pairs ΔinlA_n_fw_1_SalI/ΔinlAB_2_rv (617pb) and ΔinlAB_3_F/ΔinlB_R_XmaI, respectively. The two resulting fragments were then ligated using T4 DNA ligase (Thermo Fisher Scientific Inc., Waltham, USA) following digestion of pHOSS1 (Abdelhamed et al., 2015) with XmaI (New England Labs Inc., Ipswich, MA, USA) and SalI (New England Labs Inc.) according to the manufacturer’s instruction.

The pMADΔfosX plasmid for deletion of *fosX* was generated by removing the *fosX* sequence from a pMADfosX plasmid containing *fosX* with its flanking regions. The fosX fragment was generated by amplification from JF5203 with Hifi cloning (Takarabio Inc.) and purification with the NucleoSpin^®^ Gel and PCR Clean-up Kit (Macherey-Nagel, Düren, Germany) after gel electrophoresis. Both insertion of the resulting fragment into pMADfosX and subsequent deletion of the *fosX* sequence from pMADfosX to generate pMADΔfosX were performed with the In-fusion^®^ HD Cloning Plus (TakaraBio Inc.) technology according to the manufacturer recommendations. For In-fusion^®^ cloning, the amplification primer pairs pMADfosX3_F/pMADfosX4_R, pMADfosX1_F/pMADfosX2_R and pMADΔfosX1_F/pMADΔfosX2_R were used to generate pMADfosX and pMADΔfosX, respectively. pMADfosX and pMADΔfosX plasmids were Sanger sequenced (3730 DNA Analyzer, Thermo Fisher Scientific Inc., Waltham, USA) using the BigDye^®^ Terminator v3.1 Cycle Sequencing Kit (Thermo Fisher Scientific Inc., Waltham, USA). The primers used for sequencing (fosX_F, fosX_R, pMAD_F, pMAD_R, fosX_ex_F, fosX_ex_R) are listed in **Supplementary File S4**.

For generation of the pMAD_NactA100AA_SAG1_HIS plasmid, NactA100AA_SAG1_HIS was created *in-silico* using the Geneious 8.1 software (Biomatters Inc.) and synthesized and inserted into pMAD by Twist Bioscience (San Francisco, USA). Briefly, the *sag1* sequence NCLIV_033230 was codon-optimized for *L. monocytogenes* using a publicly available web-based software (http://www.jcat.de/). At the *sag1* 5’ end, the first 300 nucleotides of *actA* were added and the 3’ end was fused with a HIS tag followed by 2 stop codons. In order to ensure insertion of *sag1* into the *actA* locus, this sequence was inserted between the two *actA* flanking regions of the pMADΔ*actA* plasmid resulting in pMAD_NactA100AA_SAG1_HIS. For deletion of the HIS-tag to generate pMAD_NactA100AA_SAG1 (Map in **Suplementary File S1**) the fragment without HIS tag was amplified with the Hifi cloning system and integrated into pMAD with the In-fusion^®^ HD Cloning Plus using the primer pair SAG1HISdel_F/SAG1HISdel_R (**Supplementary File S4**).

#### Engineering of Lm-3Dx and Lm-3Dx_SAG1

In order to prevent permanent insertion of antibiotic resistance genes into the vector, all mutant strains were generated from *L. monocytogenes* strain JF5203 (NCBI Reference Sequence: NZ_LT985474.1; https://www.ncbi.nlm.nih.gov/nuccore/NZ_LT985474.1; lineage I, CC 1, ST 1, serotype 4b) by homologous recombination with either pMAD or pHOSS1 plasmid derivates (**Supplementary File S3**). The JF5203Δ*actA* mutant generated in a previous study (Henke et al., 2015) was used as parental mutant to engineer the vaccine vector. The JF5203Δ*actA/inlA/inlB/fosX* vector (Lm3Dx) was generated by sequential in-frame deletion of *inlA/inlB* and *fosX* genes from the JF5203Δ*actA* mutant by homologous recombination with the respective pMAD (Arnaud et al., 2004) and pHOSS1 (Abdelhamed et al., 2015) plasmid derivates described above. Gene deletions were confirmed by DNA sequencing of the intermediate mutants and by full genome sequencing of the final vaccine vector strain (see below).

Finally, Lm3Dx was transformed with the plasmid pMAD_NactA100AA_SAG1 (**Supplementary File S1**) to create Lm3Dx_SAG1 expressing *sag1* fused to the 300 first nucleotides of *actA* under the control of the *actA* promotor.

#### Quality Control

Fosfomycin minimal inhibitory concentrations (MICs) were determined in Lm WT and Lm3Dx using the fosfomycin E-test strip (Biomérieux SA, France) after 24h incubation at 37°C as previously described (Scortti et al., 2006). Genetic stability of Δ*actA* mutant and Lm3Dx was determined in comparison to the WT by serial passaging under non-selective conditions. Strains were cultured in 10mL of Bacto Brain Heart Infusion broth (BHI, Sigma-Aldrich, Buchs, Switzerland) and sub-cultured every 12 h at a 1/10’000 dilution into fresh BHI broth. Every 10 passages, 10µL of the medium was plated on BHI-Agar plate (Sigma-Aldrich, Buchs, Switzerland) and cultured 24h at 37°C before further passaging was done. Every ten passages until passage number 100, a colony of the plating performed on BHI-Agar was grown in 10mL of BHI broth and DNA was extracted using the DNA extraction kit (Invitrogen, PureLink™ Microbiome, DNA purification Kit). PCR of extracted DNA was performed to ensure stability of the deletion of each virulence gene (primers used are listed in **Supplementary File S4**). Insertion of the *sag1* sequence into Lm3Dx was confirmed by colony PCR using the *actA* flanking region primer pairs (**Supplementary File S4**), resulting in Lm3Dx_SAG1. Furthermore, both Lm3Dx and Lm3Dx_SAG1 were sequenced by INVIEW Resequencing (Eurofins Genomics, Constance, Germany) after the first and 100^th^ passage in BHI broth to analyze for single nucleotide point mutations (SNPs) (https://www.ebi.ac.uk/ena/browser/view/PRJEB43038).

For confirmation of NcSAG1 expression at the RNA level, Lm3Dx_SAG1 was cultured in BHI broth overnight at 37°C. The next day, RNA was extracted with the RNA extraction kit (RiboPure™ Bacteria, Invitrogen, Thermo Fisher Inc., Waltham, USA) and contaminating genomic DNA was eliminated with RQ1 RNaseFree DNase (Promega Corporation, Madison, USA). Purified RNA was then reverse transcribed into cDNA with GoScript™ Reverse Transcriptase (Promega Corporation, Madison, USA) following the instruction of the manufacturer. PCR for NcSAG1 was performed using NcSAG1_F/NcSAG1_R primers (**Supplementary File S4**). NcSAG1 protein expression in Lm3Dx_SAG1 during infection was confirmed by WB of proteins extracted from infected canine histiocytic DH82 cells. Briefly, DH82 cells were cultured to confluency in Corning^®^ Cell culture T150 flasks (Fisher scientific Inc., Waltham, USA) and infected at a multiplicity of infection (MOI) of 40. Six hours post-infection, infected cells were detached by Trypsin-EDTA (Sigma-Aldrich, Buchs, Switzerland), centrifuged and the pellet was frozen at -80°C. After thawing, cells were lysed by sonication (3 cycles of 2 minutes at 40% duty) (Sonifier 450, Branson, Danbury, CT, USA) and proteins were separated from the cell lysate by centrifugation (15.000 x g, 15 minutes at 4°C). Fifteen µg each of the unpurified protein extract, of Nc protein extract as positive control and of the empty Lm3Dx vector as a negative control, were separated by SDS-Page and electrophoretically transferred to a nitrocellulose membrane. The membrane was blocked in PBS/0.1% Tween-20/2% milk powder for 1 h at RT, and the monoclonal mouse anti-*Nc*SAG1 antibody (kindly provided by Prof. Dr. Camilla Björkman, University of Uppsala, Sweden) (Björkman and Hemphill, 1998) was applied at a dilution of 1:1000 overnight at 4°C. Membranes were washed four times with PBS supplemented with 0.1% Tween-20, and were incubated with the goat anti-mouse IgG coupled to alkaline phosphatase (AP) (Promega, Madison, USA), diluted 1:2000 in PBS-Tween-20. Following four more washes in PBS, bound antibodies were visualized by developing the AP reaction using 0.3% NBT/BCIP stock solution (Roche, Basel, Switzerland) in 0.1 M sodium chloride, 5 mM magnesium chloride, and 0.1 M Tris (pH 9.5). The enzymatic reaction was stopped by immersing the membrane in distilled water.

### *In Vitro* Safety

#### *In Vitro* Infection Assays

To check whether virulence gene deletion attenuates cellular infection of the vaccine vector Lm3Dx and whether attenuation is cell-type specific, canine cell lines representing epithelial cells (Madin-Darby Canine Kidney, MDCK) and macrophages (DH82) were infected. The day prior to infection, both cell lines were seeded in 24-well plates at densities of 0.8 x 10^5^ (MDCK) or 2.5 x 10^5^ cells (DH82) per well, respectively, and were grown in DMEM medium supplemented with 10% FCS and 50 µg/mL Penicillin/Streptomycin at 37°C with 5% CO_2_. One hour prior to infection, cells were starved in FCS- and antibiotic-free medium. Cells were then infected with Lm WT, Lm Δ*actA*, Lm3Dx and *Listeria innocua* at a MOI of 1.25. The concentration of the inoculum was determined by OD_600_ measurement on a Biospectrometer^®^ basic (Vaudaux-Eppendorf AG, Schönenbuch/Basel, Switzerland) and confirmed by plating of the inoculum on brain heart infusion (BHI) plates (Sigma-Aldrich). After 1h, cells were washed once with PBS and DMEM medium supplemented with FCS (2% for long-term infection assay or 10% for short-term infection assay) and 50 µg/mL gentamicin (Sigma-Aldrich) was added to prevent extracellular replication.

Colony-forming units (CFU) evaluation for the short-term infection assays was performed as previously reported (Rupp et al., 2015). Briefly, infected cells were washed once with PBS and then harvested in 100 µl of 0.5% Triton X-100 (Sigma–Aldrich) in distilled water (dH2O) 2, 4, 6, 24, 48 and 72 h post-infection (pi). Ten-fold serial dilutions of the lysates were plated on BHI-agar plates, and CFUs per well were calculated. At least three independent experiments were performed in triplicate.

For long-term infection assays, cells were infected as described above, but had to be sub-cultured due to cellular proliferation. At determined timepoints (2, 5, 7, 9 and 12 days pi) cells were washed once with PBS and then detached using 100 µL of Trypsin-EDTA solution (Sigma-Aldrich). Half of the cell suspension was transferred in a new 24-well plate with fresh DMEM medium supplemented with 2% FCS and 50µg/mL gentamicin for sub-culturing. The other half was incubated with 2% Triton X-100 (Sigma-Aldrich) in dH_2_O for 10 min and then plated in serial dilutions as described above. At the final timepoint (14 days pi) cells were harvested in 100 µL of 2% Triton X-100 and further processed as described above. At least three independent experiments were performed in triplicate.

#### Immunofluorescence Analysis of *In Vitro* Infection Assays

Coverslips with infected cells (DH82 or MDCK) were collected at 2, 6, 24, 48 and 72 hours pi and were fixed in 4% paraformaldehyde (PFA, Sigma–Aldrich) at 37°C for 15 min. Immunofluorescence was performed as previously reported (Rupp et al., 2015). Coverslips were incubated with *Listeria* O antiserum (BD Difco, 1:1000 in PBS-T) and phalloidin DyLight 633 (Fisher Scientific, Waltham, MA, USA, 1:500) in PBS-T with 10% normal goat serum (NGS, Agilent Technologies, CA, USA). After 1 h, coverslips were washed three times with PBS-T, followed by a 45 min incubation with Alexa Fluor 488 goat anti-rabbit IgG (Life Technologies, 1:500) and DAPI (1:10,000) in PBS-T supplemented with 10% NGS. Coverslips were washed twice with PBS and finally rinsed with dH_2_O and mounted using glycergel (Dako). Specimens were imaged using a FV3000 confocal laser scanning microscope (Olympus, Hamburg, Germany) and were analyzed using FIJI (Schindelin et al., 2012).

### *In Vivo* Safety and Immunogenicity

#### Mouse Vaccination

All mouse experiments were conducted in compliance with the Swiss and European Union (Directive no. 2010/63/EU) legislation for the use of animals for scientific purposes, and animal protocols were approved by the Bernese Animal Welfare Committee (licenses BE113/19 and BE103/20). For the experiments, 6-week-old female and male BALB/c as well as synchronized pregnant mice were obtained from Charles River Laboratories (Sulzfeld, Germany). All mice were maintained under conventional day/night cycle in a temperature-controlled room excepted for the mice used in the milk excretion experiment, where the cages were kept under a sterile hood after vaccination, with water and food *ad libitum*. Two weeks upon arrival at the facility, mice were randomly distributed into groups and individually marked. One additional week of acclimatization was then given to the mice prior to start of the experiment.

For each experiment inoculation doses of Lm3Dx_SAG1 or WT strain were prepared by culturing a colony overnight in 10 mL BHI broth at 37°C. Bacteria were washed 3x with sterile PBS (Sigma-Aldrich) to remove BHI-broth. Prior to injection, they were resuspended to an OD_600_ of 0.8 and sequentially diluted to the desired concentration in each experiment (1 x 10^6^, 5 x 10^6^ and/or 1 x 10^7^ CFU/50 µL). Concentration was confirmed by plating of the inoculum on BHI-Agar plates (Sigma-Aldrich) and CFU counting 24 h after incubation at 37°C. Euthanasia was performed in a euthanasia chamber using isoflurane and CO_2_ or cervical dislocation for pups younger than 3 weeks old.

Experiment 1: Biosafety evaluation of Lm3Dx_SAG1 in non-pregnant BALB/c female mice.

Mice were randomly allocated into experimental groups of 5 to 6 mice each, with an additional mouse per group used as sentinel. Three groups were injected i.m. with 50 µL sterile PBS containing the Lm3Dx_SAG1 vaccine strain at different dosages (1 x 10^6^, 5 x 10^6^, 1 x 10^7^ CFU), one group with the WT strain (1 x 10^7^ CFU) and the negative control group with 50 µL sterile PBS. Sentinel mice were used to evaluate Lm transmission between mice in the same experimental cage. Mice were immunized i.m. in the thigh. Lm3Dx_SAG1 groups received a primovaccination and two booster injections, both two weeks apart, and were euthanized 2 weeks following the last immunization (**Table 1**). Because the group receiving WT (1 x 10^7^ CFU) had to be euthanized 1.5d after the first immunization due to severity of clinical signs, 5 additional mice were inoculated i.m. with 50 µl PBS containing 1x10^7^ Lm3Dx_SAG1 and euthanized 1.5d after inoculation to assess the attenuation of our Lm3Dx_SAG1 vaccine strain during the acute phase. At euthanasia, liver, spleen, kidney, uterus, brain, thigh musculature and GIT of the Lm3Dx_SAG1 vaccinated mice were retrieved for further evaluation.

Experiment 2: Biosafety evaluation of Lm3Dx_SAG1 in lactating BALB/c dams and suckling pups.

To assess Lm3Dx_SAG1 excretion in milk and the safety of its use in lactating dams, we randomly allocated 14 dams with their respective pups (2-8 days old) into two groups (7 per group). Based on the results of the first experiment, the Lm3Dx_SAG1 group was injected once with 1 x 10^7^ CFU of Lm3Dx_SAG1 and the other group with PBS in the thigh musculature. From day 1 to day 4 after inoculation, dams were separated from their pups 1 hour prior to milking once a day (**Table 1**). Mice were anesthetized with isoflurane and 1 IU of oxytocin (Sigma) was injected intraperitoneally. Ten minutes later, milk collection was started using a custom-made vacuum pump-based milk collection system. Upon milking, dams were returned to their pups in a cage under a sterile hood until the following day. Dams and pups were euthanized at day 4 pi. The liver, spleen, thigh musculature and GIT of the Lm3Dx_SAG1 vaccinated dams and their pups were retrieved for further evaluation.

Experiment 3: Assessment of potential effects of Lm3Dx_SAG1 on pregnancy outcome in BALB/c mice.

The experiment included three experimental groups of 6 mice each. Two groups were inoculated three times at two-week intervals with 50 µl PBS containing the Lm3Dx_SAG1 vaccine strain at 1x10^6^ and 1x10^7^ CFU respectively, while the control group was inoculated with 50 µL sterile PBS at each vaccination (**Table 1**). The first vaccination was done at day 0 and the first booster on day 14. On day 22, mice were estrus-synchronized using the Whitten effect (Whitten, 1957) and were mated from day 25 to 27 by housing two females and one male in the same cage. Subsequently, males were removed from the cage and the third immunization was performed at day 28 (early pregnancy). Data on fertility (number of female mice that became pregnant), litter size (number of delivered pups per dam), stillborn mice and neonatal mortality (number of dead pups from birth until day 2 postpartum (p.p.)); postnatal mortality (number of dead pups from day 3 p.p. to the end of the experiment) and clinical signs that occurred during the observation phase were recorded. Dams and surviving pups were monitored at least 21 days after birth to rule out possible adverse effects due to treatments. At euthanasia, liver, spleen, injected thigh musculature and GIT of the Lm3Dx_SAG1 vaccinated dams and PBS control group were retrieved for further evaluation.

#### Microbiological Assessments

Mice in experiments 1 and 3 were monitored for fecal shedding as follows. In experiment 1, fecal sampling (100 mg/mouse) and analysis was performed daily from the day prior to the first vaccine administration until day 7 pi and at day 10 pi. Then, the same sampling scheme was repeated for the two booster injections (**Table 1**). In experiment 3, fecal samples of every mice were retrieved 1, 2, 4 and 7 days after each vaccine dose administration and at day 11 and 14 after the third vaccine administration.

Fecal samples were homogenized in Half-Fraser broth (Fisher Scientific) at a 1:10 dilution using 5 mm glass beads (Sigma-Aldrich) in a TissueLyser II (Quiagen, MD, USA). Ten-fold serial dilutions were plated on ALOA Agar plates (Biomérieux, France) and incubated 2 days at 37°C prior to quantification of CFUs. Additionally, bacteria were enriched by culturing fecal homogenates for 24 h at 37°C in Half-Fraser broth followed by plating on ALOA Agar Plates for qualitative evaluation. Ten-fold serial dilution of milk probes were also plated on ALOA Agar plates and enriched in Half-Fraser broth as described for fecal samples. Bacterial isolates were confirmed by phenotype on ALOA Agar plates (round, blue-green colony with an opaque halo) and colony PCR amplification with the primer pairs hly_F/hly_R, actA_F/actA_R, inlA_F/inlA_R, inlB_F/inlB_R, fosX_F/fosX_R and *Nc*SAG1_F/*Nc*SAG1_R (**Supplementary File S4**). CFU sensitivity was set at 10^3^ CFU/gram feces or milk for the quantitative assay without prior enrichment and at 10^2^ CFU/gram feces or milk for the qualitative assay after enrichment in Half-Fraser broth followed by plating on ALOA Agar plates.

For assessment of bacterial intra-host spread, organs of female mice (experiments 1-3) and pups (experiment 2) were collected under sterile conditions immediately after euthanasia to evaluate the presence of Lm in organs. Organs were divided for immersion-fixation in 4% formaldehyde (for subsequent histological and immunohistological analysis) and for homogenization and enrichment in Half-Fraser Broth (1:10) as described previously for fecal analysis. Additionally, in experiment 3, pups which died in the postnatal phase (before endpoint analysis), were weighted and homogenized in whole in Half-Fraser broth (1:10) using an ULTRA-TURRAX^®^ Tube Drive (Faust AG, Schaffhausen, Switzerland) and serial dilutions were plated on ALOA plates with and without prior enrichment in Half-Fraser broth as described above for qualitative and quantitative evaluation. At endpoint analysis, three weeks after birth, spleen, part of the liver and the gastrointestinal tract (GIT) of every surviving pup were pooled, weighted and homogenized 1:10 in Half-Fraser broth for quantitative and qualitative CFU evaluation as described above.

#### Histopathological Analysis

To screen for organ lesions caused by Lm WT and Lm3Dx_SAG1, formalin fixed and paraffin embedded muscle, liver, spleen, brain, kidney and uterus of adult mice used in all three experiments were stained with hematoxylin and eosin (H&E) and microscopically analyzed. In experiment 2, histopathology of spleen, liver and GIT of pups was performed. Additionally, most sections were screened for persistence of the vaccine vector by using an immunohistochemical protocol for detection of *Listeria*. Immunohistochemistry was performed as previously described (Oevermann et al., 2010) with a polyclonal rabbit antibody against Listeria (1:200, Difco Laboratories, Detroit MI, USA).

#### Assessment of the Immune Response Against NcSAG1

To investigate the cellular immune response against Lm3Dx_SAG1, spleens of female mice from the PBS and the different Lm3Dx_SAG1 groups of experiments 1 and 3 were used for splenocyte re-stimulation assays. Following euthanasia, spleen samples were immersed in 5 mL of complete RPMI medium (RPMI 1640 medium containing 1% antibiotic: 100 U of penicillin, 100 μl of streptomycin, 2 mM L-glutamine, 10% FCS and 55 μM β-mercaptoethanol). Splenocyte isolation was performed as previously described (Aguado-Martínez et al., 2016a). Following lysis of red blood cells and two washing steps with complete RPMI medium, cell suspensions containing the splenic lymphocytes were seeded in a 24-well plate at a density of 2 x 10^6^ cells/well.

*Ex vivo* stimulation of the seeded splenic lymphocytes was performed with concanavalin A (ConA; 5 μg/mL; Sigma-Aldrich) as positive control, with *N. caninum* antigen lysate (20 μg/mL) or recombinant NcSAG1 (20 µg/mL), respectively. Unstimulated splenocytes were used as negative control. Following culture at 37°C/5% CO_2_ during 72 h, medium supernatants of stimulated and unstimulated splenocytes were collected and levels of IFN-γ and IL-5 were measured in the supernatants using the BD OptEIA™ mouse ELISA kits (BD Biosciences, CA, USA) according to manufacturer’s instructions (Aguado-Martínez et al., 2016a). A Luminex instrument (Bio-PlexTM 200 system) was used to run the microtiter filter plates.

The expression and purification of recombinant NcSAG1 used for stimulation in our experiments was performed as follows. Firstly, the coding sequence of NcSAG1 was cloned in the pET151 TOPO^®^ expression construct (ThermoFisher Scientific, MA, USA). *Nc*SAG1 was expressed in BL21 Star™ (DE3) One Shot^®^ *Escherichia coli* strain and purified afterwards by Ni^2+^ affinity chromatography. For the purification of NcSAG1 Protino^®^ Ni-IDA 1000 packed columns (Macherey-Nagel, Düren, Germany) were used, and proteins were purified under denaturing conditions according to manufacturer’s instructions. Purity of NcSAG1 was confirmed by SDS-PAGE and the protein concentration was measured with a BCA Protein Assay Kit (Pierce™).

To determine the humoral response against NcSAG1 in experiment 1 blood samples were collected from the tail vein one day before each immunization and by cardiac puncture 14 days after the 2^nd^ booster, immediately after euthanasia. Blood of the WT group was sampled only prior to the first inoculation and immediately after euthanasia (**Table 1**). In experiment 3, to avoid stress of pregnant dams, blood was sampled only two days prior to the first vaccine application and following euthanasia, 42 days after the second booster. Blood of surviving pups was also collected by cardiac puncture. Anti-NcSAG1 IgG levels were determined by ELISA according to Debache et al. (Debache et al., 2008). In brief, 96-well plates were coated with recombinant NcSAG1 (100 ng/well) and incubated overnight at 4°C. The following day, plates were washed three times with washing buffer (0.05% PBS-Tween-20) before antigens were blocked with 1% bovine serum albumin (BSA) in 0.05% PBS-Tween-20 for 2h at RT. Subsequently, sera of individual mice at a dilution of 1:50 and two-fold sequential dilution up to 1:400 were added to individual wells. Following three washes, secondary antibody (goat anti-mouse IgG conjugated to AP (Promega)) was applied at a dilution of 1:2000. Development was performed with AP substrate buffer (1mg/mL). Absorbance was measured as optical density (OD) at 405 nm in a microplate reader (EnSpire™ 2300 Multilabel Reader, Switzerland). For evaluation, OD values were converted into relative index per cent (RIPC) values using the following formula [RIPC = (OD_405nm_ sample – OD_405nm_ negative control)/(OD_405nm_ positive control – OD_405nm_ negative control)*100 (Aguado-Martínez et al., 2016b). Serum of Nc infected mice was used as positive control.

### Statistical Analysis

Differences in arithmetic means of CFU between mutants and JF5203 were evaluated with the parametric student t-test and of the humoral and cellular immune responses between mice groups were compared with the non-parametric Mann-Whitney-U test. For the temporal analysis of the IgG response a one-way ANOVA repeated measure was used to compare each timepoint within each given group. 95% confidence intervals (95%CI) were determined and statistical significance was assigned as follows: P ≤.032 *, P ≤.0021 **, P ≤.0002 ***, P <.0001****. For IFN-γ and IL-5 concentrations, graphs are presented as column charts.

Pup mortality was analyzed with the Kaplan–Meier survival method. Pup survival curves were then compared by the Log-rank (Mantel–Cox) test, and differences in pup’s mortality rates between groups were assessed by the χ2 or Fisher’s exact test.

All statistical analysis were performed using the commercially available software Prism 9 (Graphpad Prism version 9.0.2 for MacOSX, GraphPad Software, La Jolla, CA, USA, www.graphpad.com).

## Results

### Lm3Dx Is Strongly Attenuated in MDCK Cells, but Behaves Similar to WT in Early Stages of DH82 Cell Infection

**Figure 1:**
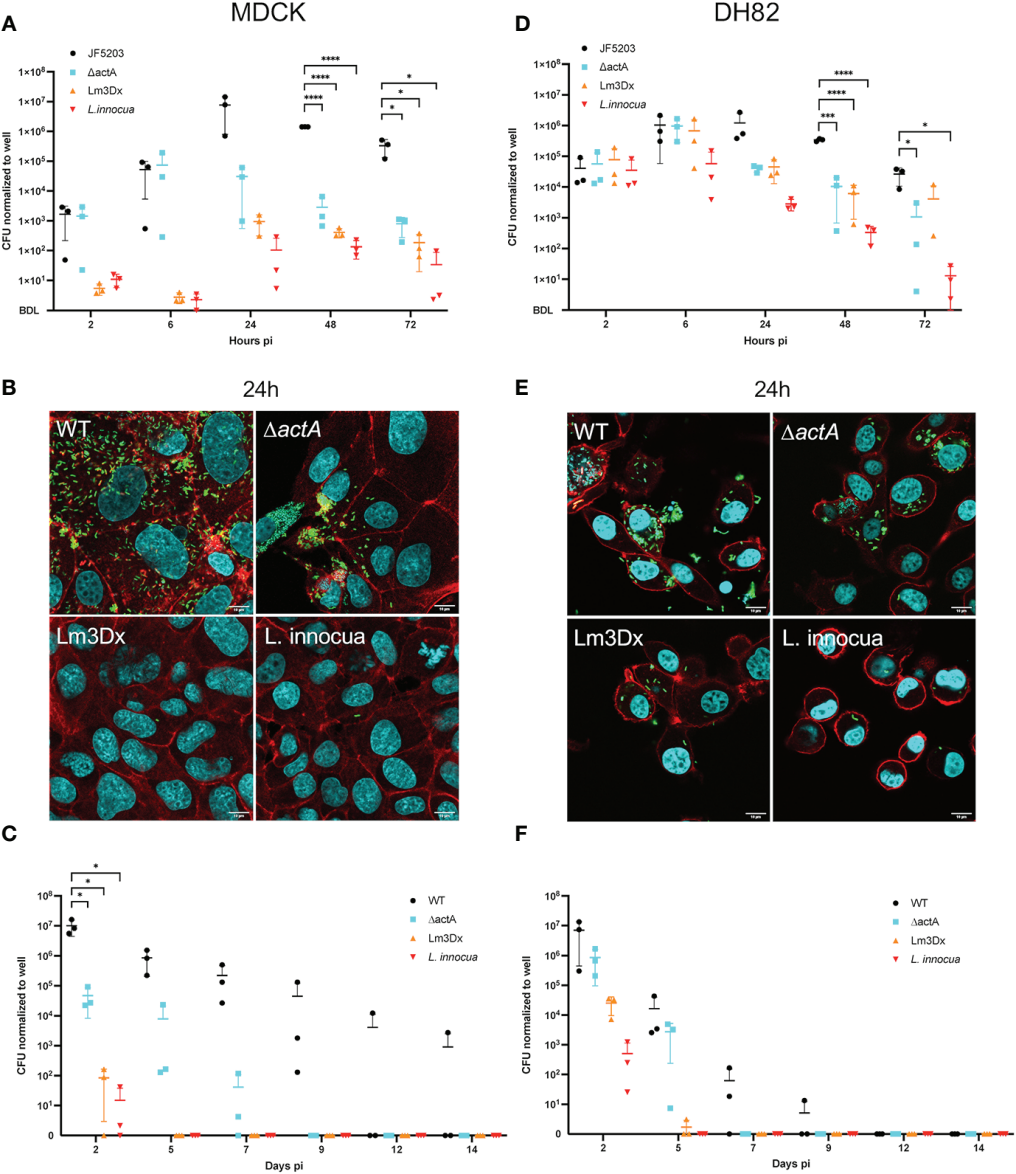
Gentamicin protection assay with the wildtype (WT) parental strain JF5203 (black circles), ΔactA (blue square), Lm3Dx (orange triangle) and *L. innocua* (red triangle) in an epithelial **(A–C)** and macrophage-like cell line **(D–F)**. For the short-term assay until 72h, canine epithelial cells (MDCK) **(A)** and canine macrophage-like cells (DH82) **(D)** were infected for 1h at a multiplicity of infection of 1.25:1. CFU were enumerated at the indicated time points. Immunofluorescence microscopy images of MDCK **(B)** and DH82 **(E)** at 24h post infection with the parental strain, mutants and *L. innocua*. Bacteria are shown in green (Listeria-Antibody), cellular actin is stained with fluorescent phalloidin (red) and cellular and bacterial nuclei with DAPI. At 24h the number of intracellular bacteria is strongly reduced in Lm3Dx-infected MDCK compared to WT-infected and ΔactA-infected MDCK, and rather similar to *L. innocua*-infected MDCK **(B)**. Additionally, Lm3Dx lacks actin polymerization, which is prominent in the WT infected cells (red comet tails). The number of intracellular bacteria is higher in Lm3Dx-infected DH82 cells **(D)** and the reduction compared to WT is less evident than in MDCK. For the long-term assay until 14d, MDCK **(C)** and DH82 **(F)** cells were infected for 1h at a multiplicity of infection of 1.25:1. At the indicated timepoints, cells were detached by trypsin and 50% of cells were transferred to a new well. The remaining cells were lysed and serial dilutions were plated for CFU counting on BHI-Agar. Lm3Dx is cleared more rapidly than the WT from both epithelial and macrophage-like cells. Datapoints in the graphs are from 3 independent experiments performed in triplicate wells and are presented with ± 95% confidence interval; *P<0.05, ***P<0.001, ****P<0.0001 (student t test). Because the y-axes of the graphs are in log scale, below detection level (BDL) was added manually to show all values where no CFU were recovered.

The parental Lm JF5203 strain (lineage I, CC1, ST1) that was isolated from a bovine rhombencephilitis case induces a strong T-cell and B-cell response (Oevermann et al., 2010). It has been extensively studied in our laboratory, and the complete genome has been published and annotated (Aguilar-Bultet et al., 2018). To evaluate the attenuation of the Lm3Dx strain and its tropism to phagocytic cells, MDCK and DH82 were used as surrogates for canine epithelial cells and APCs, respectively, and were infected with Lm3Dx in comparison to WT JF5203, Δ*actA*, and the related apathogenic bacterium *Listeria innocua* (Li) (**Figure 1**). In MDCK cells, the vaccine strain behaved similarly to the apathogenic Li and was significantly attenuated in cellular invasion compared to WT and Δ*actA* Lm as indicated by 2.5 orders of magnitude lower CFUs at 2h pi (**Figure 1A**). Confirming the CFU data, immunofluorescence microscopy detected clearly lower number of intracellular bacteria and infection foci in Lm3Dx than in WT and Δ*actA* (**Supplementary File S7**) at 2h pi. Interestingly, Lm3Dx exhibited, similar to Li, a lag phase compared to Δ*actA* and WT before the numbers of CFU raised exponentially. Additionally, Lm3Dx reached its plateau phase at a CFU count that was slightly higher than observed for Li but approximately 2 and 4 orders of magnitude lower than CFUs of *ΔactA* and WT, respectively (**Figure 1A**). The lower CFU plateaus indicated deficient intercellular spread of Lm3Dx and *ΔactA.* This was further supported by the small size of their infection foci and lower number of intracellular bacteria compared to the WT at 24h pi when analyzed with immunofluorescence microscopy (**Figure 1B**). Also, no actin polymerization was observed in Lm3Dx, *ΔactA* and Li, proving their inefficiency move intracellularly as opposed to WT and to spread from cell-to-cell (**Figure 1B** and **Supplementary File S7**). During long-term infections, the Lm3Dx vaccine strain continued to follow the infection kinetics of the apathogenic Li and was rapidly cleared in MDCK cells. CFU of Lm3Dx were detected until 2 days pi, while CFU of *ΔactA* and WT were retrieved in MDCK cells up to 7 days and 14 days pi, respectively (**Figure 1C**).

In contrast to the infection in MDCK, infection dynamics of Lm3Dx in DH82 cells were similar to the *ΔactA* mutant rather than Li. This indicates (i) the insignificance of *inlA* and *inlB* for Lm infection of phagocytic cells, and (ii) a stronger intracellular survival of Lm3Dx in phagocytes compared to Li. CFU data at 2h pi indicated that all strains entered DH82 cells with similar efficiency (**Figure 1D**) which was supported by similar numbers of intracellular bacteria and infection foci as visualized by immunofluorescence microscopy at 2h (**Supplementary File S8**). CFU numbers of Li remained constant for the first 6h and then continuously dropped (**Figure 1D**). In contrast, CFU counts of Lm3Dx increased until 6 h pi similar to WT and *ΔactA* reaching a peak of 1 x 10^6^ CFU/well (**Figure 1D**). Later, CFU of Lm3Dx and *ΔactA* dropped by approximately one order of magnitude and fewer intracellular bacteria were observed compared to the WT at 24 h (**Figures 1D, E**). Within the following 48 h, the numbers of CFUs of Lm3Dx and *ΔactA* further continued to drop, while the reduction of CFUs in the WT started only at 48 h pi (**Figure 1D**). Immunofluorescence microscopy revealed that Lm3Dx and *ΔactA* had similar number and size of infection foci confirming CFU data (data not shown). Infection foci were smaller than those in WT infected DH82 and larger than those of Li. Lm3Dx had no visible actin tails, indicating that it has lost the spreading ability (**Figure 1E** and **Supplementary File S8**). The clearance of Lm3Dx in DH82 was similar to that of the Δ*actA* mutant. While both Lm3Dx and Δ*actA* persisted in DH82 until 5 days pi, the WT was detected until 9 days pi (**Figure 1F**). Additionally, cell infection assays were performed with the intermediate mutants Δ*actA*/*fosX* and Δ*actA*/Δ*inlB*/Δ*fosX* (up to 72 h pi) and these mutants behaved as the Δ*actA* mutant in DH82 and MDCK cells leading to the conclusion that cell invasion of MDCK was attenuated by deletion of *inlA* but not *inlB (data not shown)*.

In summary, *in vitro* results showed strong attenuation of Lm3Dx in epithelial cells due to significantly reduced cellular invasion and intercellular spread, while the same strain behaved identical to the WT within phagocytic cells during the early stage of infection as further supported by the number of generations between 2-6h (**Supplementary File S5**).

### The Genome of Lm3Dx Remains Stable for 100 Passages After Deletions

**Figure 2:**
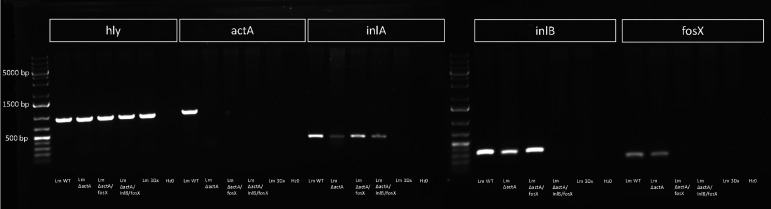
PCR for *hly, actA, inlA, inlB* and *fosX* of WT, Lm3Dx and intermediate mutants at passage 100. All strains harbor the *hly* gene encoding for Listeriolysin O that was used as species control for *Listeria monocytogenes*. Deletions of *actA, inlA, inlB* and *fosX* are stable across 100 passages.

Stability of *actA, inlA, inlB and fosX* deletions was confirmed by PCR every 10^th^ passage in BHI (data not shown) up to passage number 100 (**Figure 2**). Additionally, analysis of entire genomic sequences of Lm3Dx at passages 1 and 100 revealed that the genome was stable except the appearance of one SNP identified in the *nifJ* gene, resulting in an isoleucine substituting a methionine (G2752A) in the encoded oxidoreductase. No difference was detected in the infection phenotype of passage 100 in the tested cell lines compared to the initial Lm3Dx mutant, and likewise fosfomycin sensitivity with a MIC of 6 µg/mL compared to ≥1024 µg/mL in the WT was maintained after 100 passages (**Supplementary File S6**).

### Lm3Dx Expresses NcSAG1 at the RNA and Protein Level

**Figure 3:**
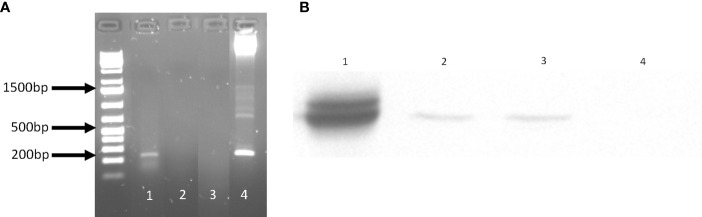
NcSAG1 expression by Lm3Dx_SAG1. **(A)** NcSAG1 mRNA expression. The 200 bp band of NcSAG1 is visible in Lm3Dx_SAG1 (1), but not in the empty vector Lm3Dx (3). H_2_0 was used as negative control (2) and pMAD_NactA100AA_SAG1 plasmid DNA as positive control (4). **(B)** Bacterial NcSAG1 protein expression in infected cells. WB evidences a specific band of NcSAG1 protein expression at 35kDa in DH82 cells infected with Lm3Dx_SAG1HIS (2) and Lm3Dx_SAG1 (3), but not in DH82 cells infected with the empty vector Lm3Dx (4). Nc crude extract was used as positive control (1).

Based on the *in vitro* results, we anticipated that Lm3Dx would not be able to cause systemic infection of epithelial organs *in vivo*, but that the early infection dynamics of Lm3Dx in phagocytes would be sufficient to trigger an efficient immune response. Therefore, as a proof of concept we created Lm3Dx_SAG1 by inserting the *Neospora caninum* sag1 sequence fused to the first 300 nucleotides of *actA* into the *actA* locus of Lm3Dx, and confirmed NcSAG1 expression in Lm3Dx_SAG1 on RNA (**Figure 3A**) and protein level (**Figure 3B**).

### *In Vivo* Safety Assessment and Immunological Characterization of the Lm3DX Vaccine Strain Expressing the Major *N. caninum* Surface Antigen NcSAG1

None of the Lm3Dx_SAG1 vaccinated mice developed adverse events or clinical signs of listeriosis after vaccination in any of the mouse experiments. In experiment 1, fecal shedding of Lm3Dx_SAG1 was detected only following enrichment in 3/6 mice from the 5 x 10^6^ CFU dose group and 1/6 mice from the 1 x 10^7^ CFU dose group at day 1 pi. Furthermore, in the 1 x 10^7^ CFU/mice group, Lm3Dx_SAG1 was recovered following enrichment at day 3 and day 7 after the first booster injection in one mouse each. In contrast to the Lm3Dx_SAG1 groups, mice injected with the parental WT strain exhibited clinical signs early on. Initial signs appeared 12 h pi and were characterized by mildly reduced general condition and slightly mat fur, while behavior remained normal. However, at 36 h pi, all mice inoculated with the Lm WT strain exhibited strongly impaired health status (isolation, lethargy, hunched posture, ruffled fur, ataxia, paralysis of the injected hindlimb) and had to be humanely euthanized. Feces of WT infected mice were positive only after enrichment in 2/6 mice one day pi, while at day 2, feces of 5/6 mice contained high numbers of bacteria (mean 4.95 x 10^6^ CFU/gram feces ± 4.76 x 10^6^ (SD)). The feces of one mouse were not recovered at day 2 pi. Feces of sentinel mice in all Lm3Dx groups and the WT group remained negative for Lm throughout the experiment.

**Figure 4:**
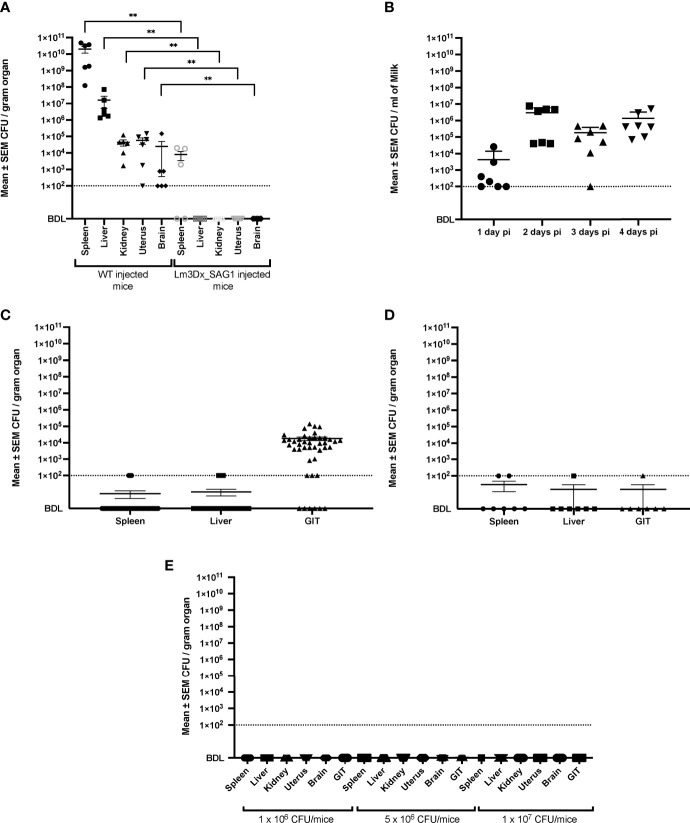
Recovery of WT and Lm3Dx_SAG1 *L. monocytogenes* from internal organs and milk. **(A)** Mean ± SEM CFU/gram organ in adult non-pregnant mice 36h following i.m. inoculation with 1 x 10^7^ WT or Lm3Dx_SAG1. Asterisks indicate significant differences to the WT group: **P ≤ 0.0021 (Mann Whitney-U test). **(B)** Excretion of Lm3Dx_SAG in milk during 4 days following i.m. vaccination of lactating dams, given as mean ± SEM CFU/ml (experiment 2). **(C)** Mean ± SEM CFU/gram organ of pups feeding milk from Lm3Dx_SAG1 dams (experiment 2). **(D)** Mean ± SEM CFU/gram organ in lactating dams 4 days following vaccination with 1 x 10^7^ Lm3Dx_SAG1 (experiment 2). **(E)** No CFU were recovered from internal organs of adult mice two weeks following the third vaccination with Lm3Dx_SAG1 (experiment 1). The data points represent individual mouse values. The detection limit for all experiments was set to 10^2^ CFU/gram organ or milk following enrichment (dashed line). BDL, Below detection limit.

After euthanasia of WT injected mice at 36h after injection, CFU count revealed high bacterial loads in most organs (**Figure 4A**). The highest bacterial loads were found in the spleen (mean 2.0 x 10^10^ CFU/gram) and liver (mean 1.62 x 10^7^ CFU/gram), while in kidney (mean 4.34 x 10^4^ CFU/gram), uterus (mean 5.72 x 10^4^ CFU/gram) and brain (mean 2.64 x 10^4^ CFU/gram) bacterial numbers were lower (**Figure 4A**). In contrast, at the same timepoint post inoculation, Lm3Dx_SAG1 was recovered only from the spleen of 3/5 mice at a mean 8 x 10^3^ CFU/gram organ, 7 orders of magnitude lower than the WT (**Figure 4A**). No Lm3Dx_SAG1 was detected in any other organ 36h after injection, not even following overnight enrichment in broth (**Figure 4A**).

In experiment 2, Lm3Dx_SAG1 excretion into milk of lactating mice was followed up to day 4 post vaccination (**Figure 4B**). Bacterial numbers reached 2.96 x 10^6^ CFU/ml of milk at day 2 and remained high until day 4 (**Figure 4B**). Additionally, Lm3Dx_SAG1 was isolated in relatively high numbers from the GIT of pups at day 4 (mean 1,81 x 10^4^ CFU/gram) indicating that bacteria were taken up orally with the milk (**Figure 4C**). However, even though Lm3Dx_SAG1 was transmitted with the dam’s milk to the pups, none of the pups showed clinical signs of listeriosis. Furthermore, Lm3Dx_SAG1 generally remained restricted to the GI tract of the pups and did not cross the gastrointestinal barrier as indicated by the low incidence of bacteria in internal organs: Lm3Dx_SAG1 was found only following enrichment in the spleen and liver of only 1 and 4/43 pups, respectively (**Figure 4C**). In line with clinical and CFU data, no histopathological lesions were observed in the GIT and internal organs of pups (data not shown). Interestingly, IHC revealed only very few bacteria in the GIT lumen of 5/43 pups, which is in line with the low number of recovered CFU from the GIT. Also in lactating dams, Lm3Dx_SAG1 was isolated from single animals and at low numbers from GIT, spleen and liver at 4d post inoculation (**Figure 4D**). Importantly, at two weeks after the third inoculation (experiment 1) no Lm3Dx_SAG1 was isolated from any organ indicating that the vector is cleared during this time interval (**Figure 4E**).

**Figure 5:**
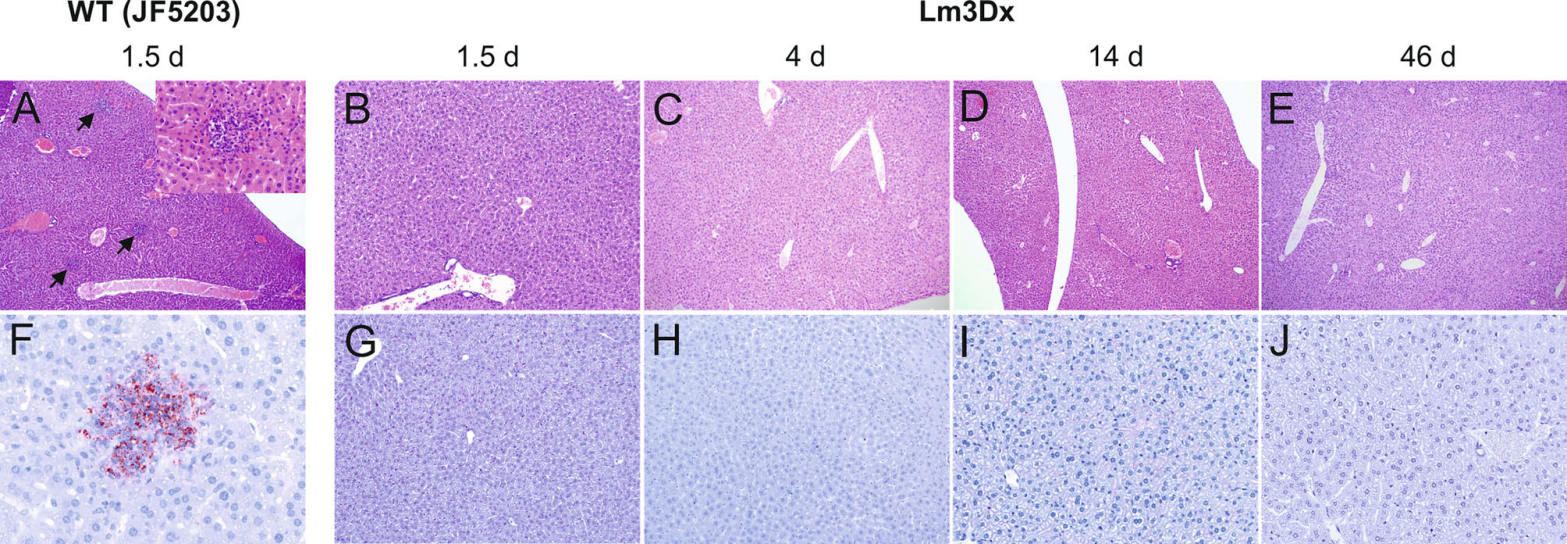
Representative HE **(A–E)** and immunohistochemically (antibody against Lm) **(F–J)** stained liver tissue of WT- and Lm3Dx_SAG1- injected mice. **(A)** Multifocal necrosuppurative hepatitis (arrows) in mice at 36h following injection with WT Lm (40x magnification). Inset: 400x magnification of a small hepatic abscess with aggregations of neutrophils obliterating hepatic cords. **(B–E)** Livers of mice injected with 1 x 10^7^ Lm3Dx_SAG1 are normal, without lesions of necrosuppurative hepatitis at all time points: **(B)** 1.5d (100x magnification) and **(C)** 4d (40x magnification) following inoculation, **(D)** 14d (40x magnification) and **(E)** 46d (40x magnification) following the second boost injection. **(F)** Necrosuppurative foci contain myriads of bacteria at 1.5d following inoculation with WT Lm (400x magnification). **(G–J)** Livers of mice injected with 1 x 10^7^ Lm3Dx_SAG1 do not contain any bacteria throughout the experiment: **(B)** 1.5d (100x magnification) and **(C)** 4d (100x magnification) following inoculation, **(D)** 14d (200x magnification) and **(E)** 46d (200x magnification) following the second boost injection.

**Figure 6:**
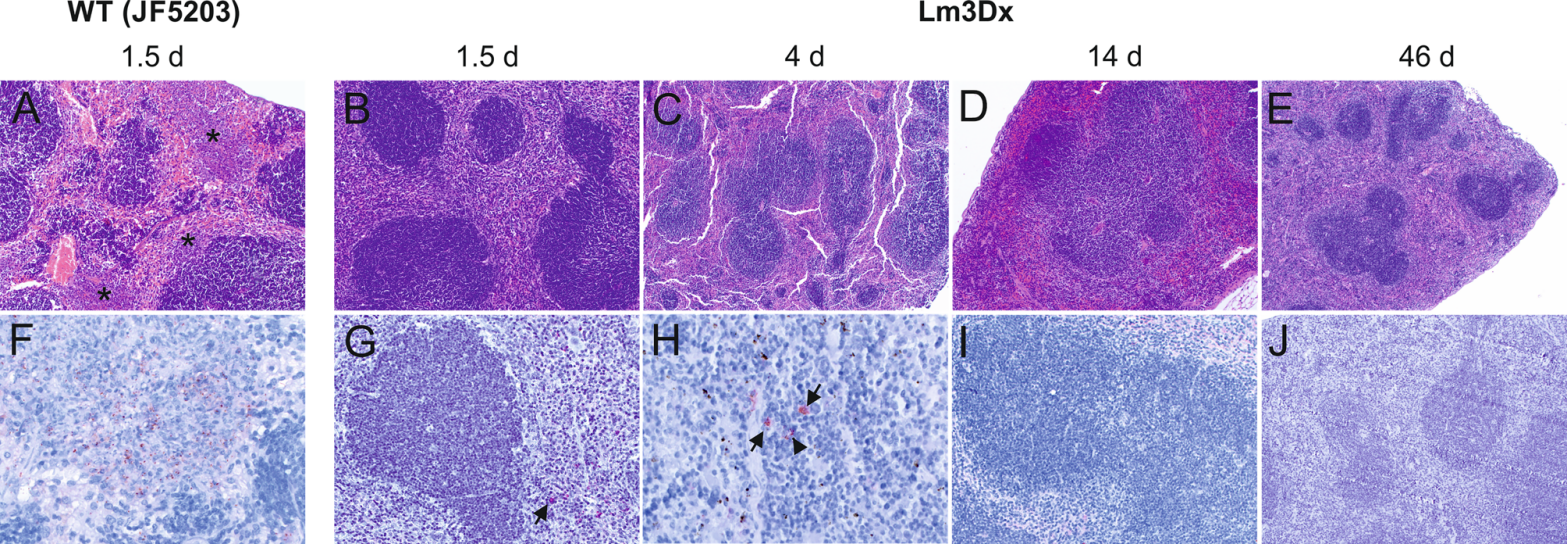
Representative HE **(A–E)** and immunohistochemically (antibody against Lm) **(F–J)** stained spleen tissue of WT- and Lm3Dx_SAG1- injected mice. **(A)** Coalescing necrosuppurative foci in the red pulp of the spleen (asterisks) in mice at 36h following injection with WT Lm (100x magnification). **(B–E)** Spleens of mice injected with 1 x 10^7^ Lm3Dx_SAG1 are normal, without lesions of necrosuppurative splenitis at all time points: **(B)** 1.5d (100x magnification) and **(C)** 4d (40x magnification) following inoculation, **(D)** 14d (40x magnification) and **(E)** 46d (40x magnification) following the second boost injection. **(F)** Necrosuppurative foci contain numerous bacteria at 1.5d following inoculation with WT Lm (400x magnification). **(G)** At 1.5d following Lm3Dx_SAG1 injection, 4/5 mice contained multifocal inflammatory cells of the splenic red pulp with a positive Lm signal (arrow), but no clear bacterial structures were observed (200x magnification). **(H)** At 4d following Lm3Dx_SAG1 injection, 4/7 lactating mice contained multifocal inflammatory cells of the splenic red pulp with a positive Lm signal (arrows), and additionally elongated positive structures compatible with bacteria (400x magnification). **(I)** At 14d (200x magnification) and **(E)** 46d (100x magnification) following the second boost injection, no positive signal was observed in Lm3Dx_SAG1 injected mice.

**Figure 7:**
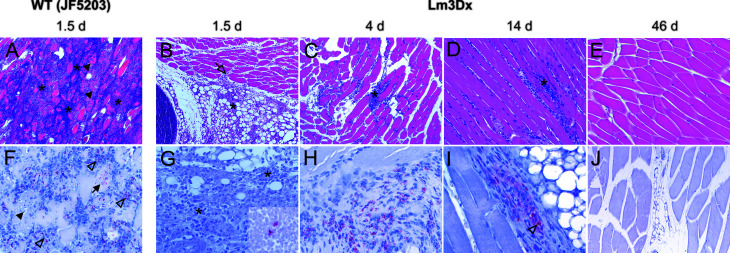
Representative HE **(A–E)** and immunohistochemically (with antibody against Lm) **(F–J)** stained muscle tissue of representative WT- and Lm3Dx_SAG1- injected mice. **(A)** Severe necrosuppurative myositis with marked neutrophilic infiltration (asterisks) and multifocal severe myofiber necrosis (arrowheads) is present in the thigh musculature of WT injected mice at 1.5d after inoculation (100x magnification). **(B)** Focal-extensive and prominent infiltration of the intermuscular fat tissue with neutrophils and macrophages (asterisk) that extends into the muscular interstitium (open arrow) in a mice at 1.5d following injection with 1 x 10^7^ Lm3Dx_SAG1 (100x). No myofiber necrosis is present. **(C)** Focal infiltrate with moderate numbers of neutrophils and macrophages (asterisk) that is restricted to the interstitial space between muscle fibers of the thigh musculature in a lactating mouse at 4d following inoculation with Lm3Dx_SAG1 (100x magnification). No myofiber necrosis is present. **(D)** Mild lymphohistiocytic infiltrates (asterisk) are present between muscle fibers of the thigh musculature of mice at 14d following the last boost with Lm3Dx_SAG1 (200x magnification). No myofiber necrosis is present. **(E)** At 46 days following the second boost with Lm3Dx_SAG1, the thigh muscle is free of inflammatory infiltrates (200x magnification). **(F)** Myriads of bacteria are observed in myofibers (arrow) and within neutrophils (open arrowheads) at 1.5d following inoculation with WT (200x magnification). Arrowhead indicates necrotic myofiber. **(G)** At day 1.5 following inoculation of adult mice, no bacteria are observed in the interstitial infiltrate (asterisks, 400x magnification). One/5 mice had a positive signal in the popliteal lymph node (inset, 400x magnification). **(H)** At day 4 following inoculation of lactating mice with Lm3Dx_SAG1, interstitial infiltrates contain moderate numbers of bacteria (400x magnification). Neighboring myofibers are normal. **(I)** Few inflammatory cells within the interstitial infiltrates contain bacteria (open arrowhead) or bacterial fragments at 14 days following the second boost with Lm3Dx_SAG1 (400x magnification). **(J)** No bacteria are present in the thigh muscle of LM3Dx_SAG1 injected mice at 46 days following the second boost (200x magnification).

The differences in bacterial load of the *Listeria monocytogenes* target organs liver, spleen, brain and uterus were reflected in the variation of pathology between WT and Lm3Dx_SAG1. All mice of the WT group suffered severe hepatitis and splenitis with necrosuppurative lesions containing large number of Listeria at 36h, while no significant lesions were present in the liver and spleen of Lm3Dx_SAG1 vaccinated adult mice at this time point (**Figures 5**, **6**). Similarly, at later time points (4 days, 2 and 6 weeks post inoculation), no lesions were observed in the target organs, independently of the physiological status of mice (lactation or pregnancy at vaccination). Immunohistochemistry revealed multifocal positivity in few scattered inflammatory cells within the spleen (**Figures 6G, H**), but not in the liver (**Figure 5**), of 4/5 and 4/7 mice at day 1.5 (experiment 1) and 4 (experiment 2), respectively. However, clear bacterial structures were only rarely present (**Figure 6H**). At later time points, no signal was observed anymore. At the site of i.m. injection, all WT inoculated mice showed at 36h large and severe necrosuppurative foci in the muscle, containing myriads of bacteria within phagocytes and myofibers (**Figures 7A, F**). In contrast, Lm3Dx_SAG1-injected mice exhibited a prominent inflammation in the thigh, which was mainly restricted to the subcutaneous and intermuscular fat tissue and occasionally extended between muscle fibers (**Figure 7B**). Myofiber necrosis, which was a prominent feature in the WT group (**Figure 7A**), was rarely observed in the Lm3Dx_SAG1 group. The cellulitis and interstitial myositis was self-limiting in the Lm3Dx_SAG1 group. At day 4 following injection (experiment 2, **Figure 7C**), all lactating mice vaccinated with 1 x 10^7^ CFU Lm3Dx_SAG1 had myositis at the site of inoculation in the thigh muscle characterized by focal to multifocal interstitial infiltrations with moderate numbers of neutrophils and fewer macrophages. At two weeks following the third injection (experiment 1, **Figure 7D**), severity of myositis was decreased consisting of mild focal interstitial lymphohistiocytic inflammatory infiltrates in 5/6, 6/6 and 6/6 mice of the 1 x 10^6^ CFU, 5 x 10^6^ and 1 x 10^7^ CFU group, respectively. By 6 weeks following the third injection (experiment 3, **Figure 7E**), the thigh muscle was free of inflammation at the injection site. Interestingly, using immunohistochemistry no bacteria were observed in the interstitial infiltrate of adult mice at 1.5d following vaccination with 1 x 10^7^ CFU Lm3Dx_SAG1 (**Figure 7G**). Only in 1/5 mouse, a positive signal was detected in a few cells of the popliteal lymph node (**Figure 7G** insert). However, when mice were vaccinated during early lactation with 1 x 10^7^ CFU Lm3Dx_SAG1, moderate numbers of bacteria were multifocally observed in the interstitial infiltrate **(** **Figure 7H**). At two weeks following the third injection, immunohistochemistry for *Listeria* revealed few bacteria and/or fragments in inflammatory cells of 4/6, 5/6 and 3/6 mice in the 1 x 10^6^ CFU, 5 x 10^6^ and 1 x 10^7^ CFU group, respectively (**Figure 7I**). At 6 weeks following the third injection, no bacteria were detected neither with immunohistochemistry (**Figure 7J**) nor with bacteriology.

The potential effect of vaccination on fertility and pregnancy outcome was evaluated and is summarized in **Table 2**. Vaccination of pregnant mice had no negative impact on the fertility or the number of viable pups. While in the PBS control group one out of 21 pups born died in the neonatal phase, 1/28 and 3/21 pups died during the neonatal phase in the 1 x 10^6^ CFU and 1 x 10^7^ CFU group, respectively (*P > 0.05*) (**Table 2**). Furthermore, vaccination of mice during pregnancy did not negatively impact the development of pups in any of the immunized groups.

**Table 2:** Data of the pregnancy outcome experiment (experiment 3). Mice received 3 doses of either 1 x 10^6^ CFU Lm3Dx_SAG1, 1 x 10^7^ CFU Lm3Dx_SAG1 or PBS.

Group	Pregnant mice	Fertility rate [%]	Litter size	Neonatal Mortality	Neonatal mortality rate [%]	Postnatal mortality	Postnatal mortality [%]	Postnatal survival [%]
10^6^ CFU	4/6	66.7	28	2/28	7.1	0/26	0.0	100.0
10^7^ CFU	3/6	50.0	21	3/21	14.3	0/18	0.0	100.0
PBS	3/6	50.0	21	1/21	4.8	0/20	0.0	100.0

### Lm3Dx_ SAG1 Triggers a Th1-Biased Immune Response in Both the Non-Pregnant and Pregnant Mouse Model

**Figure 8:**
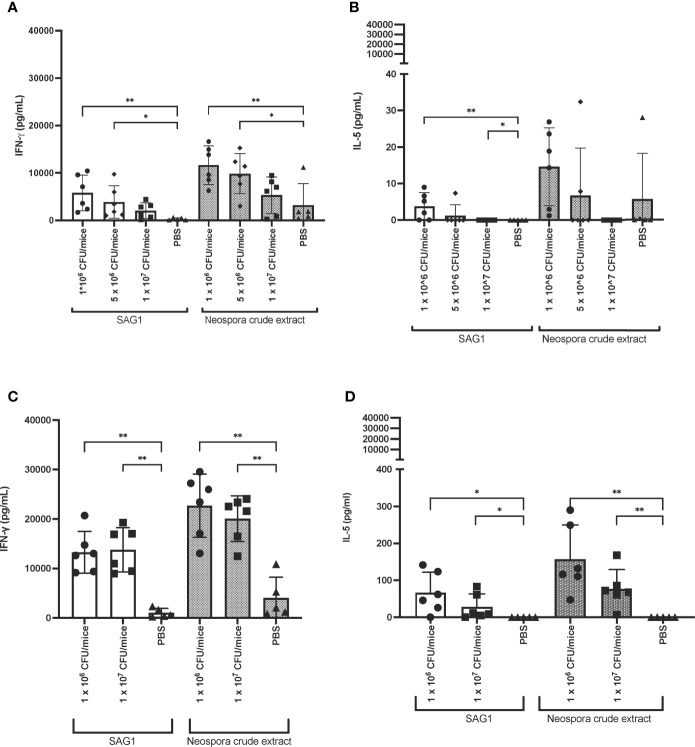
IFN-γ and IL-5 response in Lm3Dx_SAG1-immunized mice. **(A)** IFN-γ and **(B)** IL-5 response of splenocytes isolated from spleens 14 days after the second boost of non-pregnant mice. Splenocytes were stimulated with recombinant NcSAG1 or Neospora caninum crude extract (grey bars). **(C)** IFN-γ and **(D)** IL-5 response upon stimulation in the pregnancy outcome model. In this experiment, splenocytes of mice were recovered 42 days after the second boost or sham (PBS) injection. Columns represent the median and whiskers the standard deviation. * indicate significant levels compared to the PBS group: *P ≤ 0.0332, **P ≤ 0.0021 (Mann Whitney-U test).

Vaccination of non-pregnant mice with Lm3Dx_ SAG1 induced a strong Th1 biased immune response when compared to the PBS control group. Comparison to the WT group was not possible, because all WT mice had to be euthanized within 36 h after the first inoculation. We found higher IFN-γ production in splenocytes isolated from spleens of the 1 x 10^6^ and 5 x 10^6^ CFU of Lm3Dx_ SAG1 vaccinated group compared to the group inoculated with PBS (**Figure 8A**). The IFN-γ response was negatively correlated with the inoculation dose, being strongest in the low-dose group (1 x 10^6^ CFU) and lowest in mice vaccinated with 1 x 10^7^ CFU. In the latter group, the IFN-γ response was not significantly different to the PBS control group (**Figure 8A**). IFN-γ production was variable between individuals of each group with responses ranging from 5 x 10^3^ to 1.5 x 10^4^ pg/mL. The Th2-cytokine IL-5 was produced at lower levels than IFN-γ, but levels of the 1 x 10^6^ CFU/mice group were significantly higher than the PBS group (**Figure 8B**).

In the pregnancy outcome model (experiment 3), stimulated splenocytes isolated from spleens of both, dams and non-pregnant mice, produced increased IFN-γ. No difference in IFN-γ response was observed between pregnant and non-pregnant animals (**Figure 8C**). Similarly to experiment 1, IL-5 levels were increased in Lm3Dx_ SAG1 vaccinated mice compared to the PBS group, albeit at much lower amounts than IFN-γ (**Figure 8D**). For unknown reasons, cytokine concentrations were higher than in the first experiment. Factors that may have contributed to the variation might include 1) sample freezing and thawing in experiment 1, which may have led to protein degradation; 2) time point differences: blood samples in experiment 3 were collected three weeks later than in the first experiment, and in this time window the cellular immune response may have further matured; 3) inter-experimental variation.

### Lm3Dx_ SAG1 Elicits a Low-Titer Nc Specific IgG Response in Both Non-Pregnant and Pregnant Mice

**Figure 9:**
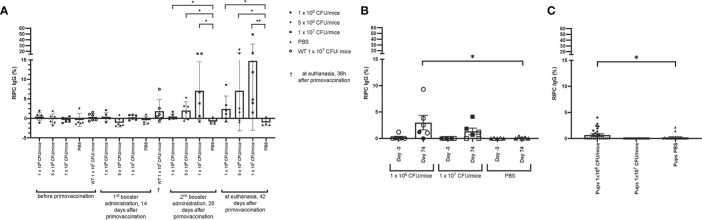
Antibody titers in Lm3Dx_SAG1-immunized mice. **(A)** NcSAG1 IgG in Lm3Dx_SAG1-immunized non-pregnant mice (experiment 1; 1 x 10^6^, 5 x 10^6^ or 1 x 10^7^ CFU/mice dose groups). Naïve mice (PBS) were included as negative control for each time point of measurement. **(B)** NcSAG1 IgG response in Lm3Dx_SAG1-immunized mice in the pregnancy outcome model (experiment 3; 1 x 10^6^ and 1 x 10^7^ CFU/mice dose groups, empty triangle, square or circle = non-pregnant mice, filled triangle, square or circle = pregnant mice). Naïve mice (PBS) were included as negative control for each time point of measurement. Sera were taken 3 days prior to primary vaccination (Day -3) and 42 days after the 2nd booster administration (74 days following primary vaccination, Day 74). **(C)** NcSAG1 IgG response in pups born from Lm3Dx_SAG1-immunized dams in the pregnancy outcome model (experiment 3; 1 x 10^6^ and 1 x 10^7^ CFU/mice dose groups). Sera were taken three weeks after birth during euthanasia and compared to sera from pups born from PBS vaccinated dams. Results are expressed as the mean of RIPC (relative index per cent) in relation to the positive control (serum of Nc infected mice), and error bars represent the standard error of the means (S.E.M) in each group. *P ≤ 0.0332, **P ≤ 0.0021 (Mann Whitney-U test).

Lm3Dx_SAG1 triggered only low titers of NcSAG1 specific IgG. In experiment 1, when compared to the baseline titers of animals before vaccination, a significant increase of IgG titers was only observed following the second booster in the 1 x 10^7^ CFU group (**Figure 9A**). Compared to the PBS group, NcSAG1-specific IgG titers were significantly higher in Lm3Dx_SAG1 vaccinated animals of all dose groups from day 28 following primovaccination (**Figure 9A**). However, titers remained low compared to the positive control (2-15% of IgG titer in serum of Nc infected animals) and similar as the IFN-γ response, titers were variable between individuals.

Also in the pregnancy interference test (experiment 3), a mild increase of NcSAG1 IgG antibody titers was observed, but as opposed to experiment 1 only in animals following vaccination with 1 x 10^6^ CFU/mouse (2.5% of IgG titer in serum of Nc infected animals, **Figure 9B**). The mean antibody titers that were determined in the 1 x 10^6^ CFU groups in experiment 1 and 3 were similar (2.4 ± 3.4 and 2.7 ± 3.7 respectively) but differed in the 1 x10^7^ CFU groups (14.7 ± 17.8 and 0.6 ± 2.6, respectively). Although the difference in antibody titers between offspring from dams vaccinated with 1 x 10^6^ CFU/mouse and from non-vaccinated dams was significant, the titers were very low compared to titers of Nc-infected animals (**Figure 9C**). In summary, the Nc specific IgG response was very low in all vaccinated animals.

## Discussion

Over the past 20 years, a growing body of evidence has shown that Lm modulates the immune response and is highly amenable to genetic manipulation, and thus constitutes a promising vaccine vector for prevention and therapy of cancer and infectious diseases (Flickinger et al., 2018). We demonstrate the safety of the highly attenuated Lm vaccine vector Lm3Dx_NcSAG1, which is devoid of any exogenous antibiotic resistance gene, in adult mice and provide first evidence of its immunogenicity.

Notwithstanding the claimed safety of previously developed Lm-based vaccine vectors with either single (Δ*actA*, Δ*prfA*) or double (Δ*actA*/Δ*inlB*) virulence gene deletions, several reports have indicated sporadic side effects post-vaccination, and isolated cases of vaccine-induced acute and delayed listeriosis have been described in humans and dogs (Maciag et al., 2009; Sacco et al., 2016; Denham et al., 2018; Fares et al., 2019). Indeed, even though *prfA* is the regulator of most of the virulence genes, *inlA* and *inlB* are also regulated by *sigB* (Kim et al., 2005), which may explain why the Δ*prfA knock-out* mutant used was not fully attenuated and may have been able to cause adverse effects upon infection. Therefore, we created an antibiotic-resistance-free triple deletion mutant devoid of *actA*, *inlA* and *inlB* to minimize the likelihood of systemic infection. Deletion of *actA* causes a 1000-fold LD_50_ increase of Lm *via* prevention of bacterial intercellular spread and, hence, systemic dissemination of infection, and additionally *via* promotion of autophagy (Flickinger et al., 2018; Radoshevich and Cossart, 2018; D’Orazio, 2019; Chávez Arroyo and Portnoy, 2020). Co-deletion of *actA* and *inlB* results in a safe vaccine vector by preventing hepatocyte infection and systemic spread while eliciting a potent immune reaction upon injection (Brockstedt et al., 2004; Flickinger et al., 2018). To further increase safety, we deleted two additional genes in Lm JF5203 generating the vector Lm3Dx: *inlA* to prevent infection of E-cadherin expressing tissues (Lecuit et al., 1997) and *fosX* to increase fosfomycin-sensitivity *in vitro* and *in vivo* (Scortti et al., 2018). *In vitro* fosfomycin resistance is an inherent feature of Lm and genetically encoded by *fosX* (Scortti et al., 2018). Therefore, *in vitro* sensitivity to fosfomycin can be used as a rapid phenotypical marker to discriminate the Lm3Dx vaccine vector lacking *fosX* from naturally occurring Lm. Moreover, *fosX* deletion further increases fosfomycin sensitivity of Lm *in vivo* (Scortti et al., 2018), and, therefore, fosfomycin becomes a potent alternative antibiotic to the commonly used ampicillin-gentamicin combination for elimination of potentially persisting bacteria after completion of the vaccination protocol and for therapy in case of post-vaccinal infection (Maciag et al., 2009; Basu et al., 2018; Safran et al., 2018).

Our *in vitro* results provided sufficient evidence of i) low risk of undesired systemic infection and persistence due to impaired epithelial cell infection and ii) sufficient APC targeting, to continue to test the vector safety *in vivo*. Additionally, we investigated the immune response against NcSAG1 in these experiments as a proof of concept by expressing the highly immunogenic *Neospora caninum* tachyzoite antigen in Lm3Dx. In contrast to most existing Lm vaccine platforms that rely on integration plasmids for antigen complementation (Lauer et al., 2002; Zebertavage et al., 2019), the gene coding for NcSAG1 was inserted by homologous recombination for two reasons. First, we wanted to avoid introduction of an exogenous antibiotic resistance gene. Although the use of integration plasmids allows rapid insertion of exogenous genes, the entire plasmid sequence containing antibiotic resistances genes allowing for selection is integrated into the vector genome at a predetermined site and retention in the bacterial genome requires propagation of the vaccine vector in selective media containing antibiotics or other selective components (Lauer et al., 2002). In contrast, when using suicidal homologous recombination plasmids, the antibiotic resistance gene is removed from the vaccine strain with the plasmid at the end of the cloning process. Furthermore, the use of a Lm vector lacking *fosX* such as Lm3Dx allows the complete abandonment of exogenous antibiotic resistance genes during cloning with recombination plasmids containing *fosX*. Secondly, we aimed to ensure insertion of the *sag1* gene into the *actA* locus, thus allowing for strong antigen expression *in vivo* to be regulated by the natural *actA* promotor, which is strongly activated by *prfA* during *in vivo* infection (Radoshevich and Cossart, 2018). Additionally, the gene integration is stable and does not require growth on selective media for maintenance. While others used homologous recombination plasmids for complementation (Frankel et al., 1995; Friedman et al., 2000), they did not insert the target antigen into the *actA* locus.

Throughout all *in vivo* studies employing the Lm3Dx_SAG1 vaccine, neither clinical signs nor adverse effects were observed, which was in contrast to WT injected mice. This is in accordance with the previously reported LD_50_ of 10^8^ CFU in a Δ*actA*/Δ*inlB* mutant (Brockstedt et al., 2004). Additionally, only few injected mice shed Lm3Dx_SAG1 in the feces, and only at a very low level. Furthermore, no lesions were detected in Lm target organs such as spleen or liver, and the local inflammation at the injection site in the thigh muscle resolved by itself within 6 weeks following the last injection as observed in histology and immunohistochemistry. Furthermore, transmission of Lm3Dx_SAG1 to sentinel mice was not observed. Altogether, these results suggest that Lm3Dx_SAG1 is not shed to a significant level into the environment *via* feces and does not persist in the immunocompetent host, which is of importance in the view of reported intravacuolar persistence and delayed infections following vaccination (Kortebi et al., 2017). Our observations including the extremely low shedding into the environment, absence of pregnancy interference, absence of clinical signs and target organ lesions as well as absence of persistent bacteria 42 days after the last boost indicates that the Lm3Dx vaccine vector exhibits a high safety level in adult non-lactating mice and, therefore, can be tested in other animal species including ruminants and dogs, susceptible hosts for *Neospora caninum*. It was surprising that Lm3Dx_SAG1 was excreted at considerable number into the milk when given to lactating mice. However, milk shedding was not associated with any clinical signs in neither dams nor pups, even though Lm3Dx_SAG1 was taken up by suckling pups with the milk. Crossing of the GIT barrier and colonization of internal organs was rarely observed in pups and only at very low levels. In line with these results, no histopathological lesions were observed in internal organs of pups. Sporadic crossing in some pups could be due to increased permeability of the GIT due to open tight-junctions at young age (Duffy, 2000; Nguyen et al., 2001; Gleeson et al., 2021). Alternatively, Lm3Dx_SAG1 might have reached internal organs *via* uptake into APCs and macrophages probing the GIT (Macpherson and Uhr, 2004). It was also surprising that while immunohistochemistry failed to detect Lm3Dx_SAG1 in the thigh muscle of non-lactating mice at 1.5d after vaccination, bacteria were still observed at day 4 in lactating mice, indicating impact of the physiological status on bacterial clearance. Milk contamination and prolonged persistence of bacteria in the muscle may be avoided by vaccinating lactating animals during the dry period or by implementing post-vaccination treatment with fosfomycin to ensure complete elimination of bacteria. However, our observations claim further investigations into mechanisms of milk excretion, impact of lactation on vector dynamics in the host and the safety of vector use during lactation before vaccination of lactating large animals is considered.

Lm infection triggers innate as well as adaptive immune responses (Wallecha et al., 2012; Flickinger et al., 2018; D’Orazio, 2019). Our preliminary data showing a strong IFN-γ production and low IgG responses point towards a Th1-biased immune response against NcSAG1. However, responses among individual mice of the same dose group varied, and not all mice developed immunity. External and internal factors such as those reported in humans (Zimmermann and Curtis, 2019) and mice (Franklin and Ericsson, 2017; Voelkl et al., 2020; Voelkl and Würbel, 2021; Zhang et al., 2021) (i.e. stress, microbiota diversity, variation in vaccination dose) may cause this variability and need to be further evaluated. Most studies showed Lm-induced immunity against foreign antigens including tumor associated antigens, HIV-1 gag, *Mycobacterium tuberculosis* and *Aeromonas hydrophila* (Friedman et al., 2000; Jia et al., 2017; Yin et al., 2017; Flickinger et al., 2018; Kim et al., 2019; Zeng et al., 2020), and they focused on the cellular immune response. One of the few studies that investigated both cellular and humoral immunity (Mahdy et al., 2019) observed rising antibody titers 14 days after the first vaccination with 1 x 10^6^ bacteria. In contrast, even though relatively low in total numbers, we observed significantly increased antibody titers only after the third dose administration (6 weeks after the first vaccination) and with tenfold more bacteria. Differences in genetic content of the engineered vectors may account for these divergences and suggest the possibility to tailor immunogenicity of Lm vectors by removal of virulence genes. In addition, the intrinsic properties of the antigens under investigation can account for these differences. The aforementioned study used a Lm Δ*actA/*Δ*plcB* Lm strain, while Lm3Dx is deleted in *actA, inlA* and *inlB*. *PlcB* is involved in listerial vacuolar escape into the cytosol upon infection (Smith et al., 1995; Quereda et al., 2018). Removal of this gene potentially increases vacuolar retention and degradation of *Listeria*, hence stimulating the delivery of bacterial proteins to the class II MHC antigen presentation machinery and enforcing the humoral arm of the immune response (D’Orazio, 2019). Future investigations will evaluate the persistence of SAG1-specific antibodies and modulation of the humoral and cellular immune responses by vector dose and deletion of further virulence genes.

## Conclusion

We here present an attenuated *Listeria* vaccine strain, Lm3Dx, which fulfills multiple safety requirements *in vitro* and *in vivo* in non-pregnant as well as pregnant BALB/c mice. However, its potential use in lactating animals needs to be further investigated. Lm3Dx is useful for stable expression of antigens of interest under the control of the *actA* promotor and is thus potentially applicable for the prevention and treatment of cancer as well as infectious diseases. Lm3Dx_SAG1 stimulates a Th1-biased immune response against NcSAG1, the major Nc tachyzoite surface protein, which is a promising neosporosis vaccine candidate. Considering the important protective role of Th1 immunity in the context of Nc infection, we suggest that Lm3Dx_SAG1 could be a potential live vaccine to be used for the prevention of neosporosis-induced fetal malformations and abortion. Thus, the safety and efficacy of the Lm3Dx_SAG1 vaccine will be further investigated in a pregnant neosporosis mouse model. In addition, since *N. caninum* is a member of the group of closely related apicomplexans including *T. gondii*, but also *Besnoitia besnoiti, Sarcocystis, Eimeria* and others, all of which impair human and/or animal health and which are bound to trigger similar immune responses (Innes and Vermeulen, 2006; Monney et al., 2011; Aguado-Martínez et al., 2017), our vaccine vector represents an interesting candidate for a wider range of applications.

## Data Availability Statement

The datasets presented in this study can be found in online repositories. The names of the repository/repositories and accession number(s) can be found below: www.ebi.ac.uk; accessions: ERS6177453 and ERS6177454.

## Ethics Statement

The animal study was reviewed and approved by Bernese Animal Welfare Committee (licenses BE113/19 and BE103/20).

## Author Contributions 

WP, AO, and PP contributed to the conception of the study. WP, AO, DI, AH, PP, NT, SG-V, and FF contributed to the experimental design of the study. WP, DI, CM, NT, and AO performed the experiments. WP performed the statistical analysis. WP wrote the first draft of the manuscript. WP, DI, AH, and AO wrote sections of the manuscript. All authors contributed to the article and approved the submitted version.

## Funding

This study was funded by the Novartis foundation (#20B089) and the Swiss National Science Foundation (SNSF) (#310030_184662).

## Conflict of Interest

The authors declare that the research was conducted in the absence of any commercial or financial relationships that could be construed as a potential conflict of interest.

## Publisher’s Note

All claims expressed in this article are solely those of the authors and do not necessarily represent those of their affiliated organizations, or those of the publisher, the editors and the reviewers. Any product that may be evaluated in this article, or claim that may be made by its manufacturer, is not guaranteed or endorsed by the publisher.

## Supplementary Material

Supplementary File S1 Plasmid map of pMAD_NactA100AA_SAG1. Image generated with Geneious (Geneious 8.1, Biomatters Inc.).

Supplementary File S2 Names and origins of bacterial strains used in this study.

Supplementary File S3 Names and origins of plasmids used in this study.

Supplementary File S4 Sequences and names of primers used.

Supplementary File S5 Number of generations during infection of MDCK and DH82 cells in the gentamicin protection assay.

Supplementary File S6 Fosfomycin sensitivity of Lm3Dx. 10µL of WT (JF5203) or Lm3Dx were grown overnight at 37°C on a BHI-Agar plate supplemented with a fosfomycin E-Stripe. The WT strain is resistant to up to 1,024 µg/mL of fosfomycin (maximum concentration tested), while Lm3Dx is sensitive to fosfomycin from a concentration of 6 µg/mL as shown by the clear halo on the agar plate.

Supplementary File S7 Immunofluorescence microscopy of MDCK cell infection with the parental WT, mutants and *Listeria (L.) innocua* over time. While the WT strain replicates efficiently in MDCK cells and clearly spreads between cells, the Δ*actA* mutant is able to invade cells, but clearly attenuated in intercellular spread. The Lm3Dx mutant and *L. innocua* are clearly attenuated in invasion efficiency with very low numbers of intracellular bacteria at 2h pi and in intracellular replication. Bacteria are labeled green, cellular and bacterial nuclei are stained with DAPI (Cyan) and cellular actin with phalloidin (red).

Supplementary File S8 Immunofluorescence microscopy of DH82 cell infection with the parental WT, mutants and *Listeria (L.) innocua* over time. In DH82, the WT, Δ*actA* and Lm3Dx are readily phagocytosed by DH82 cells and increase in numbers until 6h. While the WT shows high numbers of bacteria until 24 h and appears to decrease at 48h, numbers in the Δ*actA* and Lm3Dx mutant appear to plateau or decrease earlier. In contrast, bacterial numbers of *L. innocua* remain very low over the entire experiment. Bacteria are labeled green, cellular and bacterial nuclei are stained with DAPI (Cyan) and cellular actin with phalloidin (red).
