# Supplementary figures and images for: Safety of a Novel Listeria monocytogenes-Based Vaccine Vector Expressing NcSAG1 (Neospora caninum Surface Antigen 1)
Source: Front Cell Infect Microbiol. 2021 Aug 25;11:675219. doi: 10.3389/fcimb.2021.675219 (PMC8506043; doi:10.3389/fcimb.2021.675219)

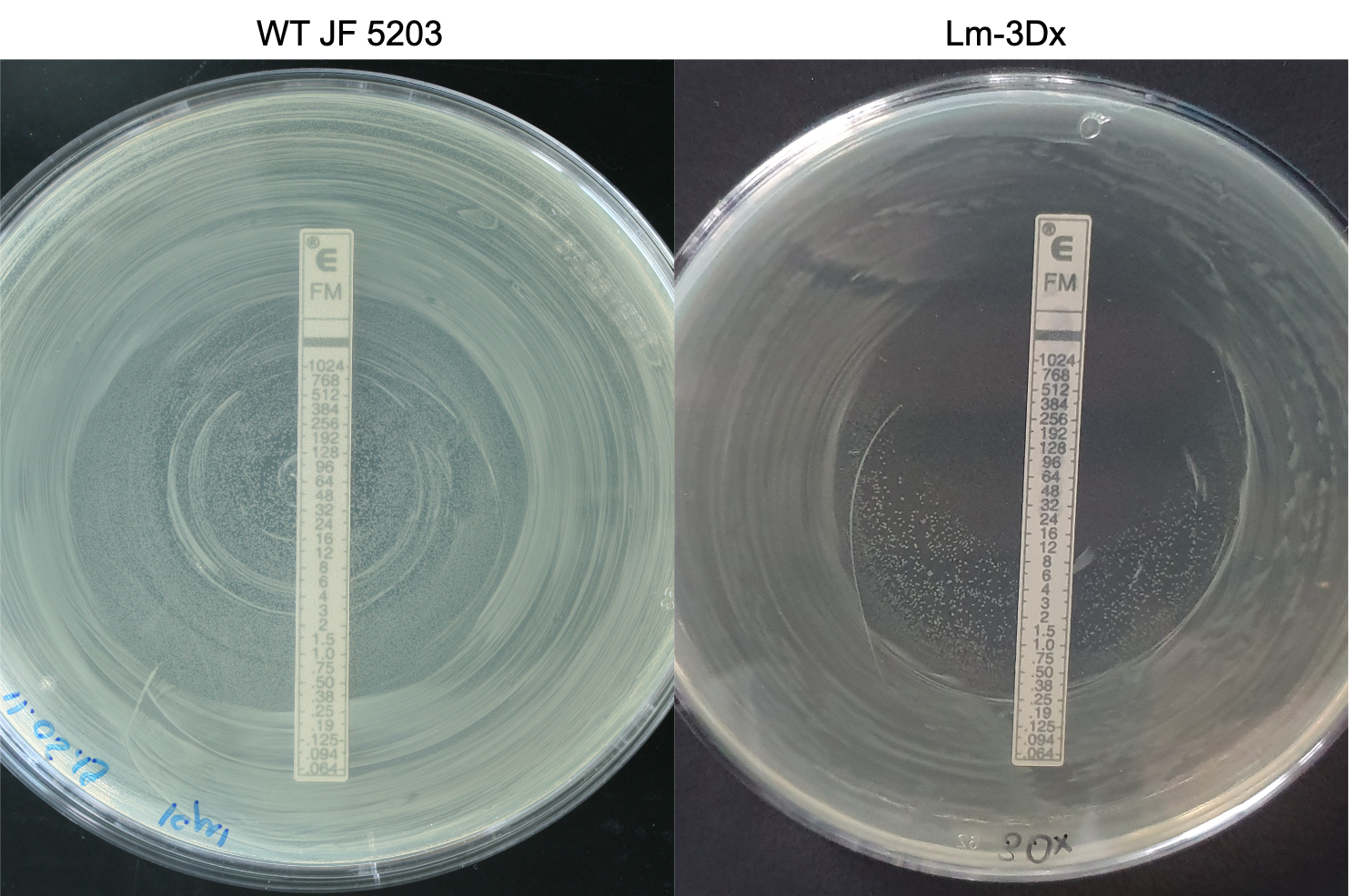

Supplement: Supplementary File S1 — Plasmid map of pMAD_NactA100AA_SAG1. Image generated with Geneious (Geneious 8.1, Biomatters Inc.). [file DataSheet_1.zip › Supplementary File S6.TIF]

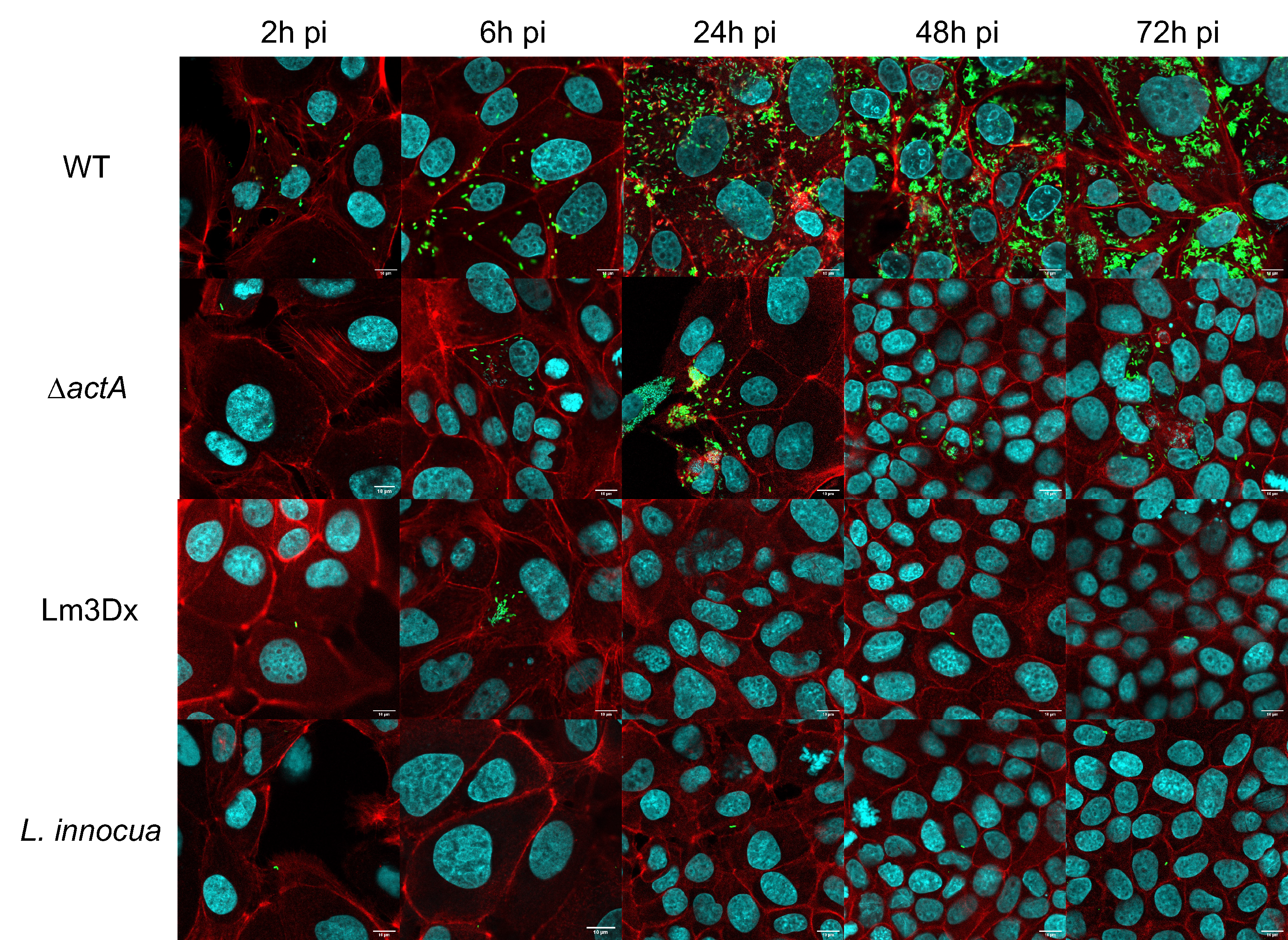

Supplement: Supplementary File S1 — Plasmid map of pMAD_NactA100AA_SAG1. Image generated with Geneious (Geneious 8.1, Biomatters Inc.). [file DataSheet_1.zip › Supplementary File S7.TIFF]

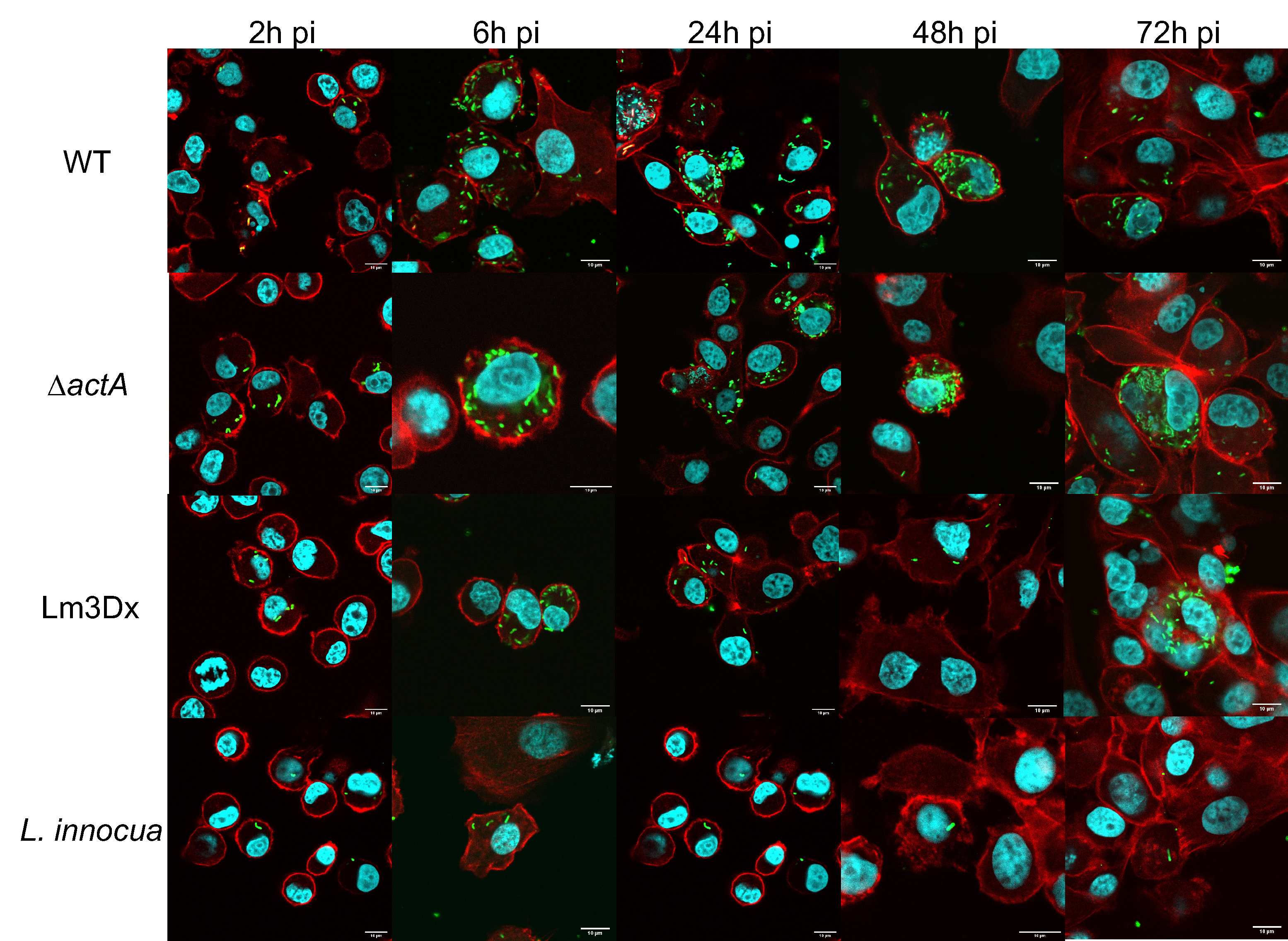

Supplement: Supplementary File S1 — Plasmid map of pMAD_NactA100AA_SAG1. Image generated with Geneious (Geneious 8.1, Biomatters Inc.). [file DataSheet_1.zip › Supplementary File S8.TIF]
